# A computational approach for the functional classification of the epigenome

**DOI:** 10.1186/s13072-017-0131-7

**Published:** 2017-05-15

**Authors:** Francesco Gandolfi, Anna Tramontano

**Affiliations:** 1 Department of Physics, Sapienza University of Rome, Piazzale Aldo Moro 2, 00185 Rome, Italy; 2 Istituto Pasteur Italia - Fondazione Cenci Bolognetti, Viale Regina Elena 291, 00161 Rome, Italy

**Keywords:** Chromatin profiles, Epigenetic mark combinations, NMF

## Abstract

**Background:**

In the last decade, advanced functional genomics approaches and deep sequencing have allowed large-scale mapping of histone modifications and other epigenetic marks, highlighting functional relationships between chromatin organization and genome function. Here, we propose a novel approach to explore functional interactions between different epigenetic modifications and extract combinatorial profiles that can be used to annotate the chromatin in a finite number of functional classes. Our method is based on non-negative matrix factorization (NMF), an unsupervised learning technique originally employed to decompose high-dimensional data in a reduced number of meaningful patterns. We applied the NMF algorithm to a set of different epigenetic marks, consisting of ChIP-seq assays for multiple histone modifications, Pol II binding and chromatin accessibility assays from human H1 cells.

**Results:**

We identified a number of chromatin profiles that contain functional information and are biologically interpretable. We also observe that epigenetic profiles are characterized by specific genomic contexts and show significant association with distinct genomic features. Moreover, analysis of RNA-seq data reveals that distinct chromatin signatures correlate with the level of gene expression.

**Conclusions:**

Overall, our study highlights the utility of NMF in studying functional relationships between different epigenetic modifications and may provide new biological insights for the interpretation of the chromatin dynamics.

**Electronic supplementary material:**

The online version of this article (doi:10.1186/s13072-017-0131-7) contains supplementary material, which is available to authorized users.

## Background

In eukaryotes, DNA is wrapped and packaged in nucleosomes, which represent the fundamental unit of the chromatin. Each nucleosome consists of an octamer of four different histone proteins: H2A, H2B, H3 and H4. These subunits undergo several types of chemical modifications on their N-terminal chain, including phosphorylation, methylation and acetylation. It has been shown that posttranslational modifications of histone proteins can modulate the structural and functional properties of the chromatin and may be associated with transcriptional activation or repression [1], suggesting that they play a key role in determining the genetic profile of distinct types of cells. However, understanding which molecular mechanisms and epigenetic changes are involved in the control of the gene expression still remains a challenge [2, 3]. Moreover, experimental evidences clearly suggest that many epigenetic modifications do not act as isolated signals along the DNA but tend to co-occur in a range of combinatorial patterns that can demarcate distinct functional elements on the genome [4].

**Fig. 1:**
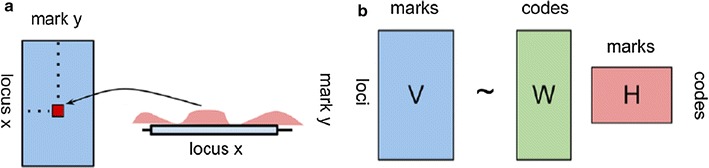
Non-negative matrix factorization of epigenetic data. The scheme gives an intuitive representation of how NMF can be used to approximate a multivariate epigenetic signal in a pre-defined number of signal patterns. The algorithm takes as input a data-matrix (V) with rows corresponding to a series of genomic intervals (or loci) and columns corresponding to different epigenetic tracks for the marks. Each cell in the matrix defines the normalized/background corrected signal of a given epigenetic mark (y) in a given locus (x) (**a**). As result, a standard NMF procedure yields two sparse matrices W (the weight matrix) and H (the coefficient matrix) describing the contribution of each code/profile to single loci and single marks respectively (**b**)

In the last decade, advanced functional genomic techniques and NGS sequencing (ChIP-seq, DNase-seq) have allowed large-scale mapping of histone modifications and other epigenetic marks, highlighting functional relationships between chromatin states and transcriptional activity. As genome-wide approaches became popular, broad datasets of different type of epigenetic data started being collected in public databases. Nowadays, two major consortia, the ENCODE (The ENCyclopedia Of Dna Elements) project [5] and the NIH Roadmap Epigenomics [6], are acquiring data on multiple types of genomic assays, including DNA methylation, histone modifications, chromatin accessibility and TF binding profiles for specific tissues/cell lines providing powerful resources to investigate several aspects of the chromatin organization. With the expanding amount of chromatin data publicly available, an increasing interest in developing computational methods able to integrate different types of epigenetic signals and identify biologically meaningful combinations of chromatin marks has emerged. Most of the proposed algorithms are based on unsupervised classification techniques aimed at identifying recurrent patterns of chromatin modifications from a given set of chromatin marks. One of the early methods, ChromaSig [7], attempts to identify commonly occurring chromatin signatures in a pre-defined set of signal-enriched loci using a pattern-finding algorithm and unsupervised clustering techniques. The approach was initially applied to nine different epigenetic marks on 1% of the human genome [8] and identified epigenetic signatures that strongly correlate with distinct types of promoters and enhancers. However, the procedure is restricted to all regions with high levels of epigenetic modifications and does not allow a full exploration of all mark co-occurrence. Other popular tools such as ChromHMM [9], Segway [10] and EpicSeg [11] partially overcome this limit providing integrative models to extract combinatorial patterns from multiple genomic experiments. All these algorithms rely on the relatively new concept of chromatin segmentation. In this approach, a genome is fully partitioned in non-overlapping segments of a fixed length and raw reads are assigned to segments (or bins) generating a count-based distribution for a given functional genomic assay. The process is repeated for each epigenetic mark generating genome-wide normalized signals of multiple genomic tracks along the chromosomic coordinate. Epigenetic signals are then processed through an unsupervised learning algorithm to infer the most probable chromatin state in each interval (i.e., a recurrent pattern of a given combination of marks). ChromHMM and EpicSeg are very similar and employ multivariate hidden Markov model to reconstruct the sequence of hidden states given a vector of observed frequencies (epigenetic marks). In ChromHMM, read count distributions are first converted in binned data tracks to reflect the presence/absence of a particular mark in each segment according to a sample-specific probabilistic threshold. This HMM approach is computationally efficient, but does not allow a full-scale analysis of the chromatin modification levels and therefore loss of quantitative information is unavoidable. In their work, Mammana et al. [11] propose a slightly different version of the HMM segmentation algorithm where raw read counts are directly used as observation variables instead of binary values. Observed counts for each epigenetic mark in each state are then modeled by a negative multinomial distribution to take into account overdispersion of the data. Segway is conceptually close to ChromHMM and EpicSeg, but employs a dynamic Bayesian networks model to infer the most probable sequence of chromatin states at 1-bp resolution. Despite their applicability, these segmentation algorithms still suffer from a number of practical limitations that make chromatin state analysis not trivial. First, in most of these methods the number of chromatin states is arbitrarily fixed a priori to allow biological interpretation of the results. This solution is convenient in practical terms but does not allow estimation of the optimal number of states for a given set of epigenetic marks. Second, most of the existing approaches are still based on computationally intensive algorithms and in the absence of an adequate compute cluster management system are hardly applicable. In this work, we propose a different computational approach to explore biologically meaningful interactions between epigenetic marks and identify a number of patterns that can be used to provide a genome-scale interpretation of the chromatin function. Our approach is based on NMF (non-negative matrix factorization), an unsupervised learning technique originally employed to approximate high-dimensional datasets in a reduced number of meaningful components [12–14]. A distinguishing feature of NMF compared to other methods is that sparse matrices of nonnegative entries are used to represent the output of the factorization (Fig. 1). This allows a better interpretation of the results and a more local representation of a given combination of marks, making the approach particularly suitable for count-based distributions as in next-generation sequencing data analyses [15]. In this study, we test the feasibility and the performance of NMF in finding recurrent combinations of marks and provide a computational framework for their full characterization. We also investigate the biological role of chromatin profiles by examining their correlation with current genomic annotations, experimental data and association with gene expression level. We first describe the preprocessing pipeline implemented to collect and integrate different types of genomic datasets for a given list of epigenetic marks and next illustrate the application of the NMF technique to identify the different chromatin profile distributions. We also qualitatively and quantitatively compare the NMF procedure to other chromatin segmentation approaches.

## Methods

### Collection of the ChIP-seq and DNase-seq datasets

We applied NMF on multiple genomic datasets collecting 13 different epigenetic marks, including ChIP-seq assays for multiple histone modifications [6, 16, 17], transcription factor binding [16, 18, 19] and chromatin accessibility assays [6] in human embryonic stem cells H1 (hESCs). All ChIP-seq and DNase I-seq experiments are part the NIH Roadmap Epigenomics Mapping consortium [6] and the ENCODE project database [5].

### Data integration and preprocessing

Each of the genomic dataset consisted of an experiment with two or more biological replicates for a given chromatin mark. In some cases, experiments from multiple laboratories for the same mark were provided. In order to generate a uniform epigenetic signal for each type of mark, we combined data from replicates in each experiment or multiple experiments (laboratories) when present.

Read alignment files (BAM or BED files) were selected from the NIH Roadmap Epigenomics data portal and the ENCODE project database and downloaded from GEO (Gene Expression Omnibus) [20].

To maintain a uniform, standard treatment of the data across different experiments, we adopted a common protocol of data processing. For each genomic assay, read alignments were processed through an *ad hoc* pipeline to generate a mark-specific normalized coverage track along the genomic coordinate.

The first part of the pipeline takes raw BAM files as input and extracts high-quality alignments generating a processed BED file for each separate sample. More specifically, we apply the following steps:

1. Remove all duplicate sequences using ‘MarkDuplicates’ function from Picard Tools (http://broadinstitute.github.io/picard)
2. Remove all reads with ambiguous matches
3. Extend unique-match reads to 200 bp in the 3′direction on both strands (this corresponds to half of the estimated average length of typical ChIP-seq fragments and is not applied to chromatin accessibility assay data)

For some datasets, processed BED files were already available and did not require any of the aforementioned steps for the final signal estimation.

For each chromatin mark, processed BED files were then combined together generating a multivariate distribution of different epigenetic signals, which represent the input to the NMF. This procedure was carried out following a standard segmentation approach. First, we partitioned the genome (hg19/GRC37 assembly version) in 200-bp non-overlapping intervals (bins), which better approximates the average occupancy of a single nucleosome along the DNA. For each sample, uniquely mapped reads were re-distributed to intervals according to their alignment positions: each read overlapping with an interval was assigned to the interval. Following the approach of Hoffman et al. [10], raw counts were then converted into background-corrected coverage estimates to account for technical and experimental variability across samples and datasets from multiple laboratories. We determined this coverage estimate as a fold-enrichment of the observed read count over the expected number of reads falling in a given bin. Specifically, we computed for each sample (replicate) *i* in the dataset *P* for the epigenetic mark *k* and the bin *j*:

- *R*(*j, k, i*), the observed number of reads of *k* assigned to *j*
- *E*(*j, k, i*), the expected number of reads of *k* assigned to *j* assuming a uniform distribution of reads over all uniquely mappable sites on the genome.

To take into account the different sequencing depth across samples *i* of *P* and the different dataset sizes, we also estimated a scaling factor which normalizes *R*(*j, k, i*) on the basis of the sample size and the average library size of the dataset. Hence, the expected read count *E*(*j, k, i*) is given by:

1 $$ E\left( {j,k,i} \right) = \frac{M\left( j \right) }{   G*Qi,P   } $$

where *M*(*i*) is the number of uniquely mappable positions in the genomic interval *j*, *G* is the uniquely mappable size of the human genome and *Q*(*i*, *P*) is the normalization factor estimated for sample *i*, defined as *Q*(*i*, *P*) = *A*(*P*)/*C*(*i*), where *A*(*P*) is the mean total count of mapped reads across all samples *i* in the dataset *P* and *C*(*i*) corresponds to the total number of mapped reads in *i*. The hg19 uniqueness mappability track was generated as part of the ENCODE project and downloaded from the UCSC Browser database [21]. Finally, a normalized coverage estimate for each epigenetic mark *k* in a bin *j* can be written as:

2 $$ S_{j,k} = \frac{{\mathop \sum \nolimits_{i}^{P} R \left( {j,k,i} \right)}}{{\mathop \sum \nolimits_{i}^{P} E \left( {j,k,i} \right)}} $$

The numerator in (2) corresponds to the sum of all observed counts in the bin *j* over samples/replicates in the dataset for *k*. This value was normalized to the sum of all expected counts from all samples in the dataset. Thus, the normalized coverage signal can also be represented as:

3 $$ S_{j,k} = \mathop \sum \limits_{i}^{P} R \left( {j,k,i} \right)\times \mathop \sum \limits_{i}^{P} \left\{ {Qi,P\times \frac{G}{M\left( j \right)}} \right\} $$

This procedure yields a sparse matrix **V**(*j*, *k*) where chromatin marks correspond to columns (*k*) and rows correspond to non-overlapping bins (*j*). Hence, each cell (*j*, *k*) in the matrix reports the final coverage estimate of a given mark (*k*) in a single 200-bp interval (*j*).

### The statistical model

In order to extract meaningful combinations of marks, we first identified regions with significant levels of epigenetic signals. This was based on a number of statistical assumptions about the data. First of all, we considered a vector **Z**_**k**_ = (*x*_1_, *x*_2_, *x*_3_…*x*_n_) of coverage estimates for the epigenetic mark **k** across **n** non-overlapping intervals in the genome. Our model assumes that **Z**_**k**_ follows a negative binomial probability distribution *Φ*_**k**_ to better represent the overdispersion of count data in typical ChIP-seq experiments. We also assumed that our negative binomial distribution arose as a mixture of Poisson distributions where the Poisson mean *μ*_**p**_ is itself a random variable, distributed according to a gamma distribution **Γ** with scale parameter α = (1 − *p*)/*p* (where *p* indicates the probability) and the shape parameter **β**. We first integrated a generalized linear model to fit each mark **k** (i.e., the vector **Z**_**k**_) on a gamma family distribution and derived **β** using a maximum likelihood estimation function. Finally, we integrated the parameters **μ**_**k**_ (i.e., the mean coverage signal over all intervals) and **β** to derive the negative binomial probability function using an alternative parametrization of *Φ*_*k*_ described by the following equation:

4 $$ \varPhi_{k} (N = n) \, = \left( {\begin{array}{*{20}c} {n + \beta - 1} \\ n \\ \end{array} } \right) + \left( {\frac{\alpha }{\alpha + 1}} \right)^{\beta } + \left( {\frac{1}{\alpha + 1}} \right)^{n} $$

where *N* is the random variable and *α*/*β* are the parameters of the Poisson–Gamma mixture distribution. The procedure generates a new probability matrix **Q**(*j*, *k*) of the same size of **V** that reports for each epigenetic mark **k** in the interval **j**, the corresponding *p* value (i.e., the probability to observe a coverage of *x*(*j*, *k*) or higher in that interval) according to the negative binomial distribution. Next, we set a statistical thresholds corresponding to a tail distribution probability of 1% and select from **V**(*j*, *k*) all genomic bins with one or more chromatin marks above the threshold. This step yields a sub-data matrix **V** ^S^(*j*, *k*) of 13 different chromatin marks distributed over 833,738 genomic intervals.

### Signal transformation

To correct for variability in the signal ranges of the different epigenetic marks, we scaled all coverage tracks (columns) in an interval from 0 to 1 using a sigmoid function (5). Values were transformed such that the coverage distribution of each mark became linear up to the 95th percentile.

5 $$ X^{{\prime }} \, = \frac{2}{{1 + {\text{e}}^{{ - 2x/{\text{y}}}} }} - 1 $$

The equation in (5) represents the sigmoid function used for the signal transformation step. The *x* parameter refers to the input normalized value for a given chromatin mark in each bin, y is the 95th percentile of the distribution, while *X*′ corresponds to the new value obtain after the transformation.

### Non-negative matrix factorization

The main task of NMF is to decompose high-dimensional datasets in a reduced number of meaningful components (profiles), which approximate the original data as accurately as possible. Brunet and colleagues employed NMF in microarray data analysis to identify patterns of gene expression that clearly discriminate between different groups of samples [14]. In a recent work, Cieslik and colleagues applied NMF to multiple ChIP-seq datasets from different chromatin marks and identified epigenetic signatures associated with distinct types of promoters and enhancers in four human cell lines [15].

Here, we used an NMF-based approach to characterize the full repertoire of chromatin profiles and capture the most recurrent combinations of marks from a given set of epigenetic signals. Due to the huge variety of possible epigenetic modifications and marks, the estimation of the real number of combinatorial profiles remains an arduous task. However, as we show here, meaningful combinations of epigenetic signals can be captured and used to characterize the most important chromatin functions in the genome.

In a general NMF model, data are approximated by two factor matrices **H**(*c*, *k*) and **W**(*j*, *c*) generated from the input matrix **V**(*j*, *k*):

6 $$ V \, \approx \, W\;H $$

where **H**(*c*, *k*) represents the pattern coefficients matrix and **W**(*j*, *c*) a matrix of weights to reconstruct **V**(*j*, *k*) using the patterns described by H. In **H**(*c*, *k*), rows corresponds to signal profiles while columns corresponds to samples (i.e., the different epigenetic marks of **V**). Thus, each cell in **H**(*c*, *k*) reports the contribution of each pattern c to the epigenetic mark k. The **W** matrix has the same number of rows as **V**(*j*, *k*) and columns corresponding to the number of patterns. Hence, each cell in **W** indicates the weight of a given profile c in each genomic interval. According to (6), each column of **V**(*j*, *k*) is approximated by a nonnegative linear combination of the columns of **W** (profiles) where coefficients are indicated by the corresponding columns of **H**(*c*, *k*). A schematic representation of the NMF procedure is shown in Fig. 1.

The weights and the coefficient matrices need to be initialized with a seed (i.e., a value for *W*_0_ and *H*_0_), from which the iteration process can start. The most common seeding method is to use a random starting point where the entries of W and H are drawn from a uniform distribution over the range [0, max(**V**_*j, k*_)]. A general rule of thumb for the stochastic initialization approach is to perform several runs of the NMF (i.e., several random initializations for matrices W and H) and keep the factorization that minimizes the reconstruction error across multiple runs.

7 $$ { \hbox{min} }[\delta = V \, {-} \, WH \, ] $$

where *δ* is the difference between the real and the model output values of the epigenetic mark levels.

The most important parameter in NMF is the factorization rank *r*, which corresponds to the expected number of combinatorial profiles used to approximate **V**(*j*, *k*). As with most unsupervised learning algorithms, the choice of the optimal *r* represents a critical step in an NMF analysis and a clear consensus strategy to determine the best value of *r* is still lacking. In general, large factorization ranks results in sparse signal profiles (i.e., many patterns containing data from a single variable/mark) and few combinatorial interactions. Conversely, too small values of *r* compress the data in a scanty number of patterns where spurious interactions between epigenetic marks are more likely to arise.

A common approach to find the best factorization rank is to try NMF in a pre-defined range of *r* values, estimate a quality measure of the results, and select the best value of *r* according to this quality criteria [22].

Different strategies have been proposed to select the best factorization rank. The most common approach is based on the *cophenetic correlation coefficient*, which reflects the overall cluster stability obtained after the factorization process [14]. Furthermore, the cophenetic coefficient strongly depends on the sample–sample distances from the consensus and the connectivity matrices. Given the total number of epigenetic tracks collected and analyzed *K*, the *K* × *K* connectivity matrix gives the empirical probability for each sample pair to be part of the same cluster. In NMF, connectivity matrices over multiple runs are then averaged to derive the final consensus matrix. The cophenetic correlation coefficient is defined as the correlation between the sample distances from the consensus matrix and the distances obtained by its hierarchical clustering [14]. Brunet et al. proposed to select the value of *r* after which the cophenetic coefficient starts decreasing. A more robust approach suggests to take the smallest value of *r* at which the decrease in the residual sum of squares (RSS) between **V**(*j*, *k*) and the NMF model is larger than the decrease observed in the random data [23].

We applied NMF to the sub-data matrix of **V**(*j*, *k*) defined as **V** ^S^, which describes the normalized coverage signal of the 13 chromatin marks over a set of 833,738 genomic bins. In the same manner, we used NMF on a random data matrix **V** ^R^ having the same column and row sizes as **V** ^S^. We generated the **V** ^R^ matrix independently, by randomly permuting values in the columns of the real matrix.

The whole procedure was carried out in the R environment [24] using the NMF framework package [22]. To achieve a reasonable cluster stability, we executed, for each NMF analysis (corresponding to a given r), 30 different runs using the ‘Brunet’ algorithm [14] and a random initialization approach. In order to speedup the computation, all runs were parallelized on a 56Gb RAM multi-core machine using the *foreach* and *doParallel* framework packages.

Since the goal of NMF is to reduce the dimensionality of the original data, an appropriate factorization rank should be chosen such that *r* < min(*j*, *k*). To identify the best *r*, we first defined a range of ranks between 3 and 13 and performed multiple NMF runs on both the real and the random data matrices to compute the value of *r* in that range. A minimum factorization rank of 3 was chosen since this is the minimum *r* value previously assessed in [15] and because common histone modifications tend to coarsely accumulate in three distinct types of genomic regions: promoter, intragenic and intergenic. Hence, cophenetic correlation coefficients from both the real and the random datasets were computed and compared together for each value of *r* in the range 3–13 to identify significant changes in the cluster stability. To derive a reasonable statistics, each NMF analysis was repeated 20 times, generating a distribution of ‘random’ cophenetic coefficients for each rank. Specifically, we took the value of *r* above which the cluster stability of the real dataset started being significantly higher than that in the random (using a distance of more than fourfold standard deviation from random mean as threshold). We referred to this value, which corresponds to the optimal factorization rank chosen for the analysis, as r*. Given an NMF model with a number r* of combinatorial profiles, we finally reconstructed the full pattern membership of each feature (genomic intervals) in **V** ^S^ by assigning each bin to the profile *c* with the maximum contribution in that interval according to the weights of the **W**(*j*, *c*) matrix.

### Genomic feature annotation and gene expression data collection

Annotations for genes and other types of genomic features were downloaded from the UCSC Genome Browser database [25] using the hg19 genome assembly version (feb 2009). Specifically, we retrieved the full list of Refseq gene coordinates, together with the 3′ UTR and 5′ UTR regions, Refseq introns and exons, Refseq upstream regions (defined as 1-Kb regions before the TSS) CpG islands [26], poly-adenylation sites [27], small regulatory RNAs and microRNAs [28, 29], conserved human enhancers [30] and conserved transcription factor binding sites [31]. All Refseq transcripts from the same locus sharing a common TSS were merged together resulting in a final list of 23,086 TSS-grouped transcripts.

The Vista database [30] of human enhancers provides a list of conserved noncoding regions experimentally validated by moderate mouse transgenesis enhancer assay. From the initial list, enhancers with reproducible expression in at least three independent biological replicates (also called positive enhancers) were selected, resulting in a final set of 642 validated regions. The enhancer annotation was also integrated with a list of 684 putative hESC-specific enhancer clusters collected from the dbSUPER database [32], catalog super-enhancer regions predicted in several human and mouse tissues/cell lines from ChIP-seq experiements. The conservation data of putative TFBS were obtained from the Transfac Matrix Database [31]. The full set of TFBS was next filtered in order to keep sites with strong evidence of sequence conservation (*Z* score >2.3). hESC-H1-specific DNase hypersensitive sites (peaks) were taken from the UW (University of Washington) group as part of the ENCODE project and downloaded from the UCSC data portal. Heterochromatin region coordinates were obtained from the UCSC Broad ChromHMM track as part of a chromatin state segmentation study using ChromHMM on nine different epigenetic marks in human ESC-H1 cells [9]. 5C (Chromatin Conformation Capture Carbon Copy) chromosomic interaction data from H1 cells were generated by the Dekker Lab/University of the Massachusetts [33] and downloaded from the UCSC genome database as well.

hESC-H1 promoter expression data were obtained from UCSC as genome-wide CAGE track provided by the RIKEN consortium [34]. CAGE (5′ cap analysis of gene expression) levels were reported as RPKM (reads per kilobase per million of mapped reads) for each CAGE cluster (i.e., a region of overlapping tags assigned to a value representing the normalized expression signal). Robust CAGE clusters were finally identified by selecting all those regions with an irreproducible discovery rate (IDR) smaller than 10^−3^.

hESC-H1 gene-level expression estimates were previously generated by the ENCODE/Caltech groups through single-end RNA-seq experiments in four different biological replicates and downloaded from the ENCODE GRCh37.v3c annotation database [35]. Normalized expression values (RPKM) for each locus in each sample were averaged across all replicates to get the mean RPKM estimate of the gene.

### Selection of transcription factor binding data

Mapping positions of putative TF binding sites were taken from the Myers Lab at the HudsonAlpha Institute of Biotechnology [36]. The dataset consisted of a collection of 16 different transcription factors, each represented by two distinct biological replicates. Each sample was provided as a tab-delimited file in the ‘broadPeak’ format and contained genome-mapping coordinates of the enriched regions (peaks) detected through ChIP-seq binding assays in hESC-H1. For each transcription factor, the intersection between peak coordinates in the two different replicates was obtained using the *bedtools intersect* function and compared to the different chromatin profiles.

### Enrichment analysis of chromatin profiles

For each chromatin profile (or state), the extent of overlap with a given type of genomic feature (or transcription factor binding peaks) was assessed using a 2 × 2 contingency matrix. Let M_u,g_ be a matrix for the profile *u* and the genomic feature *g*, having **a** and **b** values in the first row and **c**/**d** in the second; **a** is the total number of bins belonging to *u* that have a minimum overlap of 1-base pair with any region from *g*; **b** is the estimate of the number of bins from *u* that do not overlap with any region of the feature *g*. We calculated **c** as the number of bins assigned to any profile *u* ^*I*^ ≠ *u* that overlapped for at least 1 bp to any region of *g*. We computed **d** as the number of bins belonging to *u* ^*I*^ that did not overlap any region of the same feature. Finally, the enrichment ratio for the chromatin profile *u* compared to the feature *g* was calculated as [*a* × *d*]/[*b* × *c*]. The significance of the enrichment was assessed using one-tail Fisher’s exact test with a *p* value threshold of 10^−5^.

### Analysis of chromatin data using different segmentation approaches

We ran NMF independently on the same hESC-H1 dataset maintaining a random initialization approach and the ‘brunet’ method as core algorithm (with 30 runs) but increasing the factorization rank from 7 to 13. The hESC dataset was also analyzed in parallel using the ChromHMM algorithm [9] as alternative approach for the comparison. Since normalized signals for these marks were already generated, we used the *BinarizeSignal* function of ChromHMM tool to convert directly the input matrix into a binarized dataset using the default statistical threshold (Poisson tail probability of 10^−4^). After the binned data were obtained, the *LearnModel* function was applied to perform chromatin state analysis on a whole-genome scale. The -*printposterior* and the -*printstatebyline* parameters were also included to retrieve the posterior probability vector over state assignment and the assigned chromatin state per bin. We ran ChromHMM in two independent analyses using either an 8-states or a 14-state model at a bin resolution of 200 bp. In this paper, we actually reefer to these analyses as the 7-states and the 13-states models since, compared to NMF, an additional “empty-state” is normally generated by ChromHMM.

### NMF analysis in IMR90 human cell line

Multiple epigenetic datasets from human IMR90 (human fetal lung fibroblasts) cell line were downloaded from the ENCODE project database [5] and the NIH Roadmap Epigenomics consortium [6] as read alignment files (BAM/BED) for the same epigenetic marks collected from hESC-H1. Each chromatin dataset was represented by a pool of two (or three) biological replicates for a given chromatin mark. For each genomic assay, a normalized coverage signal track was generated following all the steps in the implemented pipeline as previously described. Once the IMR90 normalized matrix was obtained, genomic bins were filtered assuming a negative binomial probability tail of 0.01 and all the signal distributions were compressed into a common range (0,1) using a sigmoid function for the data standardization. Next, we ran NMF on the new combined dataset using *r* = 7 and the same algorithm parameters. It is important to note that a factorization rank of 7 was set to facilitate the comparison of chromatin profile distributions between the two cell lines.

## Results

We run the NMF algorithm on the filtered data matrix **V** ^**s**^ representing the coverage signal of thirteen different epigenetic marks over 833,738 significant bins in human embryonic stem cells. The observed trend of the cophenetic correlation coefficient from both the real and random datasets is shown in Additional file 1: Figure S1. Comparison with the random data (red line) shows a first gain in stability at *r* = 4 after which the cophenetic coefficient rapidly decreases (blue line). A second increase is observed for *r* = 7. At this point, the cophenetic coefficient of the random dramatically drops reaching a minimum at *r* = 11, while that of the real dataset progressively reaches a plateau (coefficient = 0.99). In this range, the real cophenetic coefficient constantly remains at more than fourfold the standard deviation from the mean of the random dataset. Hence, we decided to select a factorization rank of 7, which corresponds to the smallest value of *r* where stability starts being significantly higher than the random.

### Chromatin profiles definition and interpretation

**Fig. 2:**
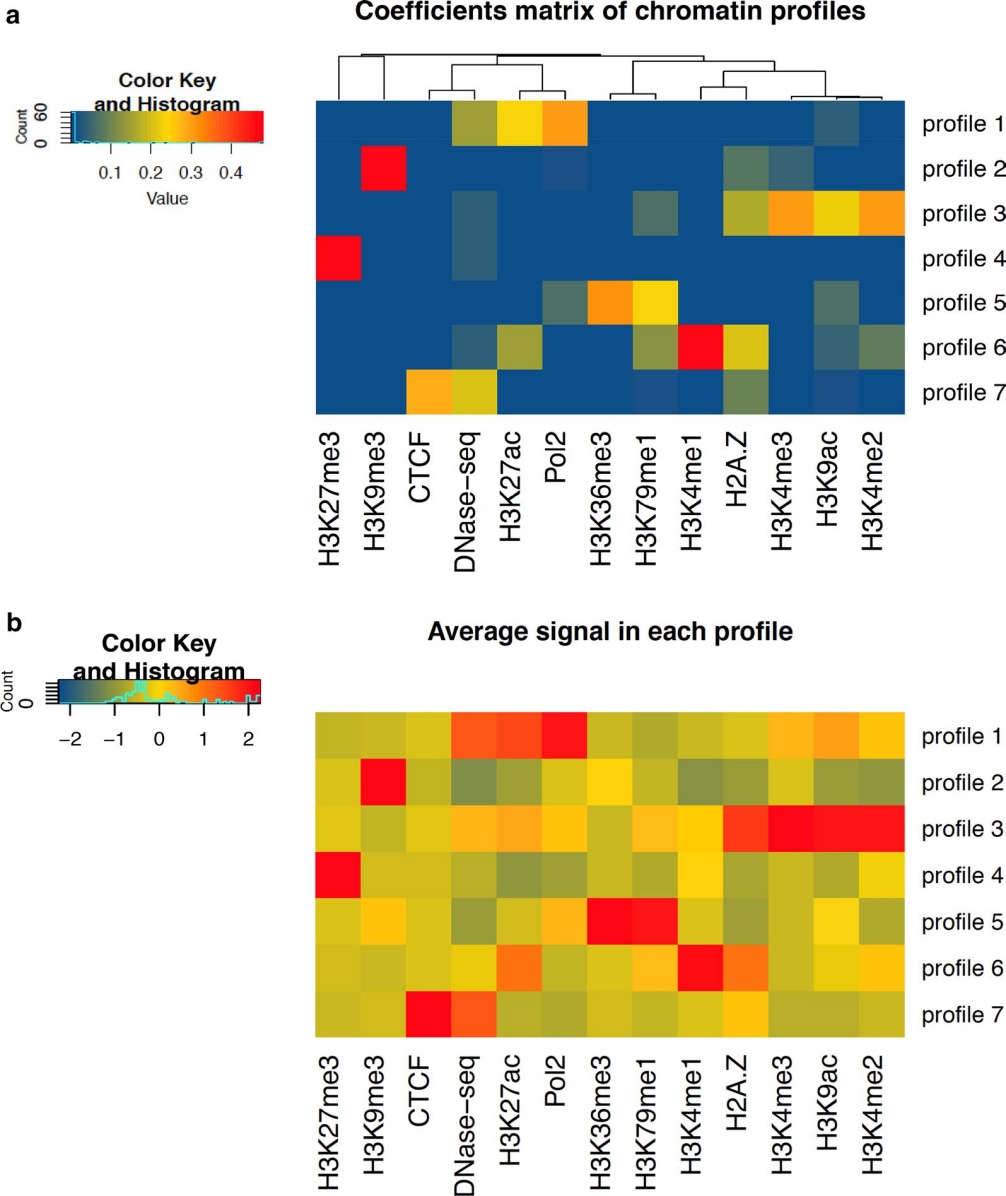
Chromatin patterns definition and interpretation. *Upper panel* **a**: color-scale heatmap showing the hierarchical clustering of 13 different epigenetic marks on the coefficient matrix H obtained with seven factorization ranks. Each cell (*x*, *y*) in the matrix indicates a pattern coefficient reflecting the contribution of the code in X to the epigenetic track defined in Y. Hierarchical clustering analysis clearly identifies different subgroups of *marks* **b**: *color-scale* heatmap and hierarchical clustering using the average normalized signal of each *mark* (*columns*) across all genomic intervals of a given profile (*rows*). Average signals are centered and scaled such that the mean of the epigenetic mark in each *column* is zero

One of the main advantages of the NMF-based approach compared to other chromatin segmentation methods is that the number of combinatorial profiles is not fixed in a predetermined manner, but effectively relies on the extent of correlation among input signals. An intuitive representation of the seven combinatorial profiles obtained after the NMF procedure is illustrated in Fig. 2.

The first matrix corresponds to the coefficient matrix (H) generated by the algorithm (Fig. 2a). The H matrix shows that each epigenetic profile is composed by a distinct pattern of marks, indicating a high level of dissimilarity and the presence of specific combination of chromatin signals. Notably, epigenetic profiles 1 and 3 cover the majority of the activator marks, suggesting an association with open promoters and transcriptionally active regions. Despite this, the two profiles differ remarkably when we look at the contribution of single marks. Profile 1 is mostly defined by a combination of two marks, H3K27ac and Pol2, with a minor contribution from the DNase HS (hypersensitive site) mark. Conversely, profile 3 reveals a completely different mark combination, which primarily consists of H3K4me2, H3K4me3, H3K9ac and a minor load in H2A.Z, a histone variant involved in the control of the promoter activity and gene responsiveness to specific physiological conditions. While the H2A.Z mark seems to be less represented, histone modifications H3K4me2 and H3K4me3 show the highest contribution to the profile. We also observe a moderate contribution of H3K9ac, a histone-acetylation mark known to be associated with active promoters. A different cluster of histone modifications is observed in chromatin profile 6. This epigenetic status is predominantly enriched in H3K4me1, which is known to be associated with distal enhancer regions [37]. However, the profile also exhibits a much more lower content of four additional epigenetic marks (H2A.Z, H3K4me2, H3K27ac, H3K79me1), which is likely to suggest a modest level of chromatin activation.

Chromatin profile 7 apparently lacks any promoter-associated or transcription-associated histone modification, but shows moderate enrichment in DNase hypersensitive sites and CTCF binding, with a minor contribution in H2A.Z. CTCF is an insulator binding protein that can interact with promoters, enhancers or other types of DNA regulatory elements, activating or repressing the transcriptional machinery according to the type of the bound DNA sequence [38]. Notably, the absence of any acetylation or methylation mark is quite consistent with the functional role of CTCF, which is prevalently found in intergenic sequences, often distant from the transcription start site. This chromatin profile is mostly represented by a combination of CTCF and DNase HS, an epigenetic mark extensively used to map active cis-regulatory DNA elements by identifying chromatin accessibility regions in the genome [18, 39, 40].

Profile 5 consists of a combination of four different chromatin marks. Among them, the most prevalent, H3K36me3, is a tri-methylated histone mark associated with RNA elongation within the body of transcribed genes. Another transcription-associated mark, H3K79me1, also appears in the same profile with a smaller contribution. Other two marks, Pol2 and H3K9ac, are mostly linked to active promoters or other regulatory regions but are more poorly represented than transcription marks H3K36me3 and H3K79me1. The last two epigenetic profiles, 2 and 4, are dominated by the presence of two different repressive chromatin marks, respectively: the histone tri-methylation H3K9me3 and H3K27me3. Profile 2 combines H3K9me3 with very low occurrence of H2A.Z, whereas H3K4me3 and other TSS/transcription-associated marks are almost absent. Chromatin profile 4 shows a high contribution of the histone modification H3K27me3, a well-characterized mark associated with polycomb-mediated repressed regions and promoter inactivation. Interestingly, we found that the chromatin mark composition of profiles 3, 4, 5 and 6 was very similar to that of some profiles (defined as epigenetic ‘codes’) previously obtained in [15] in the same cell line using a slightly different set of histone modifications.

To evaluate the robustness of each epigenetic track in each combination of marks, we also estimated the mean coverage signal of every mark across the different profiles. As shown in the heatmap of Fig. 2b, the mean signal distributions resemble well the composition of different chromatin profiles as reported in the coefficient matrix (Fig. 2a). Taken together, these results demonstrate the applicability of the NMF approach in discovering combinatorial information among multiple epigenetic marks, highlighting functional interactions otherwise not easily decipherable using separate ChIP-seq assays.

### Genomic distribution of chromatin profiles

**Fig. 3:**
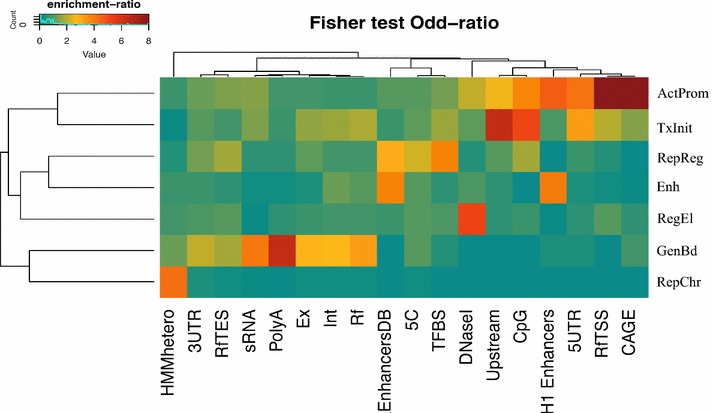
Enrichment of chromatin profiles with respect to genomic features. The heatmap represents the enrichment of each chromatin profile (*rows*) compared to different regions of the gene and distinct types of genomic features in the genome (*columns*). The enrichment is defined as a log-odd ratio as described in “Methods”. Positive associations (odd-ratio >1) are colored from *green*/*yellow* to brown whereas negative associations (odd-ratio <1) are indicated in blue. As shown in the heatmap, each combinatorial profile reveals a distinct pattern of enrichment, thus demonstrating the usefulness of the NMF-approach in the biological interpretation of the different chromatin functions. In this heatmap, each profile is associated to a specific biological label in order to facilitate the mnemonic association between the profile and its functional role on the basis of the observed enrichment (*top-bottom*): *ActProm* = Active Promoter (profile 1), *TxInit* = Transcription Initiation (profile 3), *RepReg* = Repressed Regulatory Regions (profile 4), *Ehn* = Enhancer Regions (profile 6), *RegEl* = Regulatory Elements (profile 7), *GenBd* = Gene Body Transcription (profile 5), *RepChr* = Repressed Chromatin (profile 2). Genomic features indicated in the columns are: CAGE = hESC-H1 CAGE clusters from ENCODE; RfTSS = Refseq Transcription Start Sites; RfTES = Refseq Transcription End Sites; 5UTR=Refseq 5’untranslated region; 3UTR = Refseq 3’unstranslated regions; H1 Enhancers = Superenhancer regions from hESC; CpG = CpG islands; Upstream = 1Kb upstream regions from Refseq TSSs; DNase1 = hESC DNase1 Hypersensitive sites from ENCODE; TFBS = Conserved transcription factor binding sites from the Transfac Matrix Database; 5C = Chromatin conformation capture carbon copy data from hESC; EnhancersDB = experimentally validated enhancer elements from the VistaEnhancer Dabatabse; Rf = Refseq genes; Int = intronic sequences from Refseq genes; Ex = exonic sequences from Refseq genes; PolyA = predicted poly-adenylation sites; sRNA = small RNAs; HMMhetero = predicted heterochromatin regions in hESC

As a first step, we sought to characterize each profile as a function of their genomic their distribution using a set of functional elements and well-annotated regions as benchmark. To define the biological significance of the different profiles, we compared their genomic distribution with those of a number of known functional regions, including Refseq genes, Refseq promoters, enhancers, poly-adenylation sites, 5′ and 3′ UTRs, smallRNAs, CAGE clusters, transcription factors binding sites and other types of functional genomic data (see “Methods” section). Interestingly, the overlap analysis identified very different patterns of enrichment among the profiles, indicating the presence of distinct epigenetic functions (Fig. 3; Additional file 1: Figure S2).

To provide a more intuitive and biologically interpretable definition of each profile, we replaced numbers from 1 to 7 with a list of seven distinct ‘genomic labels’ (and abbreviations) on the basis of the different enrichment patterns using the following scheme: chromatin profile 1 = ‘Active Promoter’ (ActProm); chromatin profile 2 = ‘Repressed Chromatin’ (RepChr); chromatin profile 3 = ‘Transcription Initiation’ (TxInit); chromatin profile 4 = ‘Repressed Regulatory Regions’; chromatin profile 5 = ‘Gene Body Transcription’ (GenBd); chromatin profile 6 = ‘Enhancer Regions’ (Enh); and chromatin profile 7 = ‘Regulatory Elements’ (RegEl).

The first group of combinatorial profiles, ‘Active Promoter’ and ‘Transcription Initiation,’ shows specific enrichment in Refseq promoter-associated regions. Conversely, the two profiles clearly diverge when we focus on the specific group of the most enriched features. ActProm (which reefers to the Pol2/H3K27ac/DNase HS mark combination) is heavily enriched in close proximity (±50 bp) of the Refseq TSS (fold-enrichment or f.e. = 8.1, Fisher’s exact test *p* value = 10^−10^) and hESC-H1 CAGE clusters (f.e. = 8.9, *p* value = 10^−10^), with marked spreading toward the 5′UTR of the gene (f.e. = 4.1). Notably, the profile shows moderate overlap with DNase I hypersensitive sites (f.e. = 2), CpG islands (f.e. = 3.7) and 1-Kb upstream regions (f.e. = 2.5). Similarly, profile TxInit (defined by chromatin marks H3K4me2/H3K4me3/H3K9ac) is predominantly found around promoters and the 5′end regions of the gene, but shows higher enrichment in CpG islands (f.e. = 5.11, *p* value = 10^−10^) and TSS-upstream regions (f.e. = 6.4, *p* value = 10^−10^). Furthermore, it shows moderate levels of enrichment in the 5′UTR of the gene (f.e. = 3.27, *p* value = 10^−12^), but decreases in proximity of gene transcription start sites (Refseq TSS ± 50 bp f.e = 1.97, CAGE clusters f.e = 1.5), where we observe lower overlap from fourfold to fivefold lower compared to chromatin profile 1. The TxInit profile is also associated with conserved transcription factors binding sites (f.e. = 1.62, *p* value = 10^−8^) and Refseq intragenic regions (genes f.e. = 1.9, exons f.e. = 1.55, introns f.e. = 1.62), suggesting a more spread distribution around the gene TSS. Globally, these results demonstrate that both the chromatin profiles are strongly associated with the regions surrounding gene promoters but show inverse patterns of enrichment around the transcription start site, indicating a possible different organization of the chromatin near the 5′ portion of the gene. Compared to them, the ‘Gene Body Transcription’ profile shows a completely different enrichment pattern, with a significant overlap over the body and the 3′ end of the gene (Refseq genes f.e. = 3.35, exons f.e. = 2.7, introns f.e. = 2.66, TES f.e. = 1.7, 3′UTR f.e. = 2, poly-A sites f.e. = 6.5). This profile also shows significant association with annotated small RNAs, for which it shows consistent enrichment (f.e. = 4.02, *p* value = 10^−8^), but results strongly depleted in the promoter-associated regions. Chromatin profiles RepChr, RepReg, Ehn and RegEl have very poor overlap with all promoter-related regions, but show the tendency to spread over the intergenic portions of the genome. Despite the prevalence of a repressive mark, profiles RepChr and RepReg show distinct patterns of enrichment when we look at their genomic context. While the former is mostly concentrated toward putative heterochromatin regions (f.e. = 4.2, *p* value = 10^−8^), the latter shows preferential enrichment for enhancers (f.e. = 4, *p* value = 10^−8^), conserved TF binding sites (f.e. = 3.88, *p* value = 10^−8^) and distal chromatin interactions (f.e. = 2.28, *p* value = 10^−10^), but it results weakly represented in TFBS, CpG islands and Refseq 3′-end regions, thus suggesting a degree of association with inactive or poised enhancers. Profile RegEl (the CTCF/DNase HS profile) is strongly associated with open chromatin accessibility regions (f.e. = 5.2, *p* value = 10^−10^), suggesting an association with different types of cis-regulatory DNA elements (insulators, silencers, etc.) and a role in the organization of the chromatin structure. The last combinatorial profile Ehn significantly correlated with both the enhancer groups (Vista Database Enhancers f.e. = 3.83, *p* value = 10^−8^, hESC super-enhancers f.e. = 3.9, *p* value = 10^−10^), where it shows higher levels of overlap compared to profile RepReg. Interestingly, we found that profile ActProm is also significantly associated with hESC active enhancers, with even higher enrichment than the ‘Enhancer’ profile (f.e = 4.6, Fisher *p* value = 10^−10^). It is noteworthy that, in contrast with Enh, the ActProm profile seems to be confined to active enhancers only, as suggested by the extremely poor overlap with the Vista Database annotation. This different enhancer pattern is likely to suggest that, within enhancer regions, the two profiles are often correlated and, while the presence of Ehn profile is crucial for enhancer prediction, the addition of ActProm could help in discriminating between the active and the poised enhancer state.

**Fig. 4:**
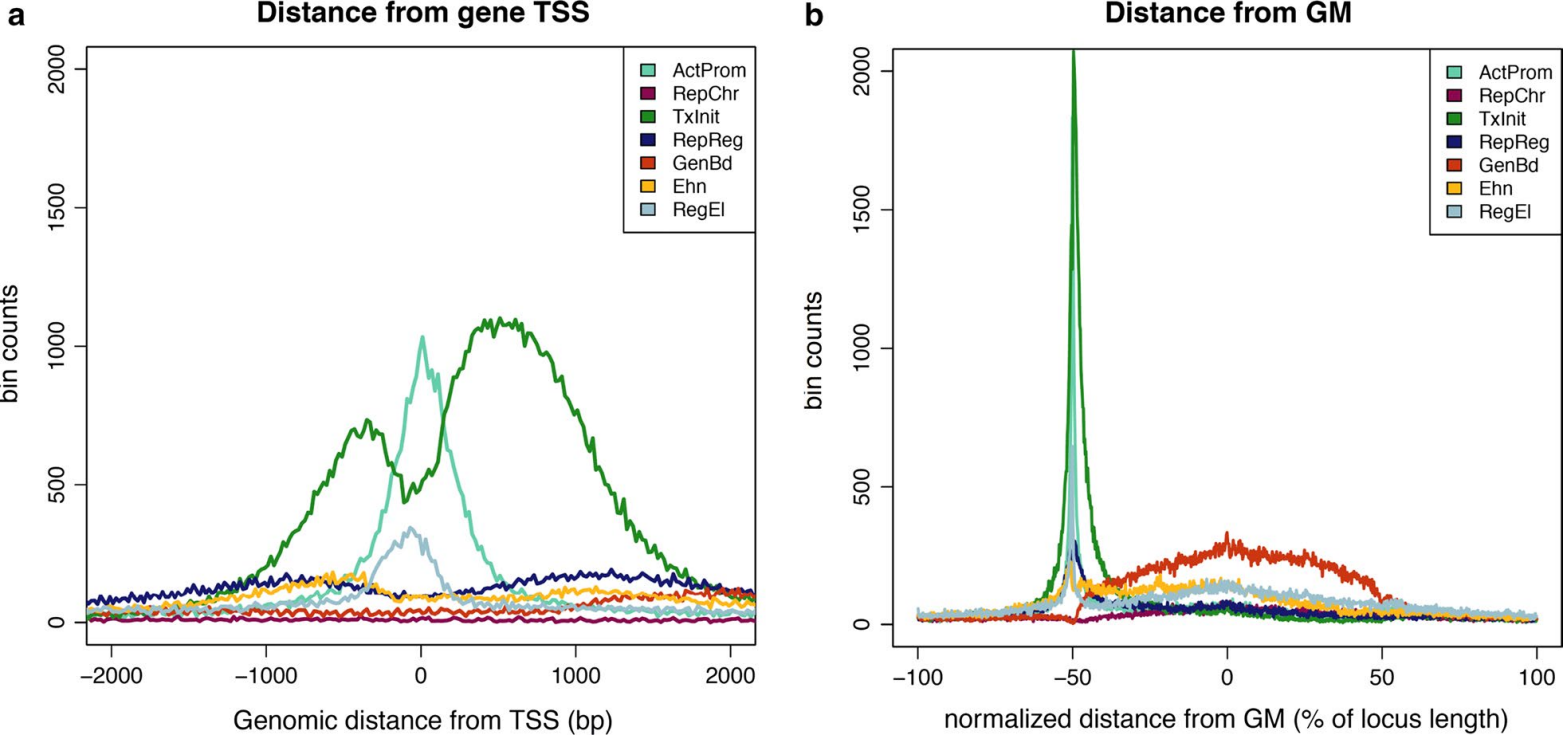
Frequency distribution of chromatin profiles around the transcription start site (TSS) and the gene middle-point (GM). The histograms plot the distribution of the different chromatin profiles around the TSS (**a**) and the central position of Refseq genes (**b**). Both distributions are generated on the basis of the observed distance (bp) of each bin to the closest TSS (corresponding to 0 on the *x*-axis) or GM. As shown in **a**, two major epigenetic profiles (ActProm and TxInit) are enriched around the promoter region of the gene. In **b** the genomic distances are normalized to the gene length so that the middle-point of the gene is always at the 0 position, the gene length is normalized from −50 to +50

To get a more detailed view of chromatin profiles occurrences in the gene structure, we also investigated how chromatin profiles are differentially distributed around specific structural elements, such as the TSS and the middle point of the gene. Hence, each combinatorial profile was analyzed with respect to its distance from the closest gene feature (Fig. 4a, b). Around the TSS, the most striking difference is observed between the two promoter-associated profiles, ActProm and TxInit. These chromatin profiles occur with highest frequency compared to all other profiles, but show completely distinct shapes over a region of 4 Kb surrounding the 5′end of the gene (Fig. 4a). While ActProm precisely maps to the transcription start site, chromatin profile TxInit tends to be broadly distributed both in the upstream and downstream directions, with two large peaks in the TSS surrounding region and a characteristic dip exactly overlapping the TSS (Fig. 4a).

To investigate spatial relationships among the different profiles, we also examined the frequency with which a given profile consecutively occurs next to each other considering all possible pairwise combinations. For any transition A–B, we estimated its occurrence as the logarithmic fold-change of the frequency observed in the real data over the frequency in the random dataset (Additional file 1: Figure S3). Notably, the analysis reveals the presence of a low number of meaningful associations. Among these, transition from chromatin profile TxInit to ActProm shows the highest level of enrichment (logFC = +1.07). The reciprocal transition ActProm-> TxInit is weakly enriched (logFC = +0.45) relative to the random profile distribution. Among the most recurrent profile combinations, transition from TxInit to Ehn seems to be also slightly favored (logFC = +0.80). Transition RegEl-> Ehn (+0.37) and ActProm-> Ehn (+0.77) also occur with higher frequency than randomly expected, but show lower occurrence relative to the most frequent profile combinations.

### Recovery power of chromatin profiles for a known set of genomic features

**Fig. 5:**
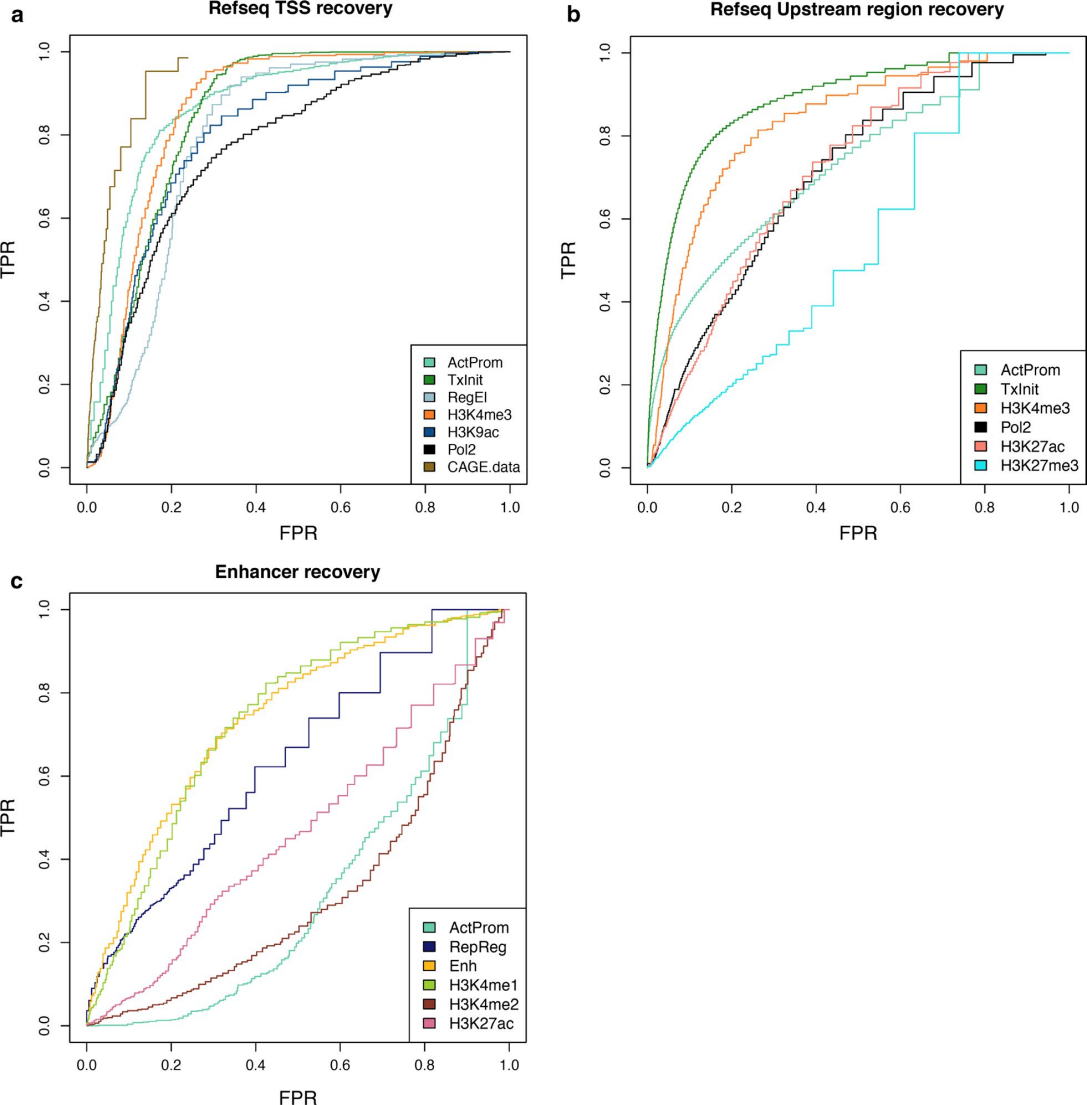
Recovery power of chromatin profiles compared to single chromatin marks. The plots show the Receiver Operating Characteristic (ROC) curve for the ability of different chromatin profiles and single marks in recovering Refseq TSSs (**a**), Refseq upstream regions (**b**) and experimentally validated enhancers (**c**)

We next evaluated the ability of the different chromatin profiles in correctly recognizing distinct classes of known functional elements and comparing their performance with that of most representative epigenetic marks. For this task, we focused on a small set of functionally relevant regions of the gene that are already supported by a consistent amount of experimental information: the Refseq TSS, the 1-Kb upstream region from the gene TSS, enhancer regions and RNA poly-adenylation sites. For each region, the predictive power of chromatin profiles was assessed using a ROC (**r**eceiver **o**perating **c**haracteristic) curve (Fig. 5a–c; Additional file 1: Figure S4). To test the performance in the Refseq TSS prediction, we focused on a list of 11,263 transcription start sites supported by hESC-H1 CAGE cluster data (i.e., Refseq promoter regions overlapping at least one CAGE cluster in a window of ±50 bp around the transcription initiation site). We found that, in almost all cases, chromatin profiles showed better performance compared to that of single chromatin marks, confirming the ability of NMF in identifying combinatorial interactions that are more informative than single mark contributions. Here, chromatin profile ActProm shows a performance similar to that of the CAGE clusters, but outperforms predictions based on TSS-associated marks (H3K4me3, H3K9ac, Pol2) and those of other mark combinations. Similarly, profile TxInit shows the best performance in correctly predicting 1-Kb regions upstream of the Refseq TSS (Fig. 5b), with higher recovery power compared to the performance of promoter-associated marks. Notably, the analysis of enhancer predictions (Fig. 5c) shows that, at relatively lower false positive rates (<0.25), the ‘Enhancer’ profile and the ‘Repressed Regulatory’ profile slightly outperform predictions from H3K4me1 mark and other related histone modifications such as H3K4me2 and H3K27ac. In contrast, no improvement was observed when we compared the performance of profile GenBd with the predictive power of the H3K36me3 mark (Additional file 1: Figure S4).

### Ambiguousness in chromatin profile assignment

**Fig. 6:**
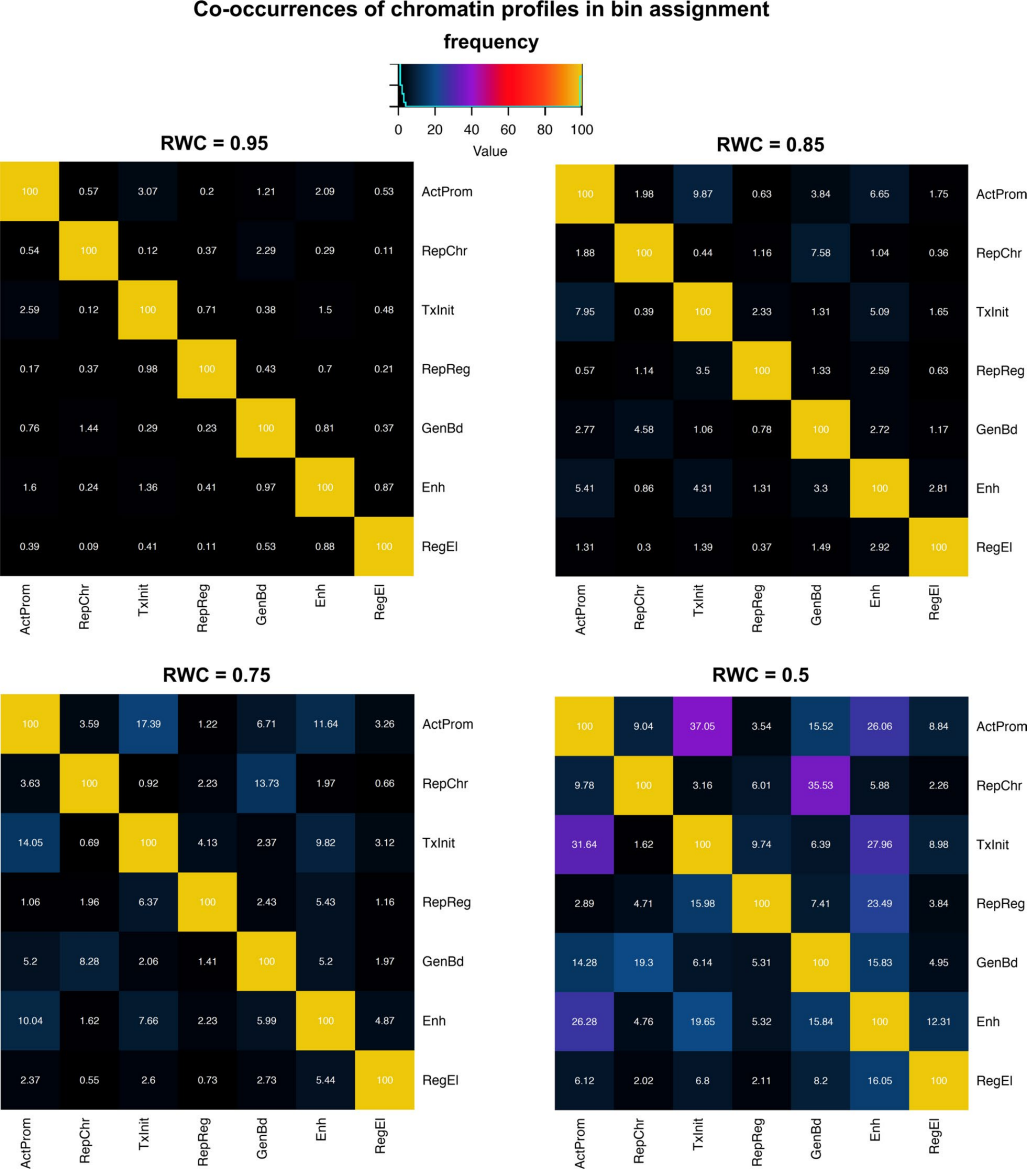
Co-occurrences of chromatin profiles in bin assignment. Each heatmap in the figure is a 7 × 7 matrix showing the frequency of co-occurrence of each profile compared to each other in all possible pairs at different RWC (Relative Weight Contribution) thresholds (0.95; 0.85; 0.75; 0.5). Chromatin profiles are reported in *either rows* or *columns* using the same labels previously adopted in the manuscript. Profiles from the original bin assignment are reported in the *rows*, whilst additional profiles (*columns*) are progressively co-assigned as the RWC threshold decreases

We also set out to assess how confidently a profile could be univocally assigned to each bin by simply taking the dominant (i.e., the maximum) weight observed in the *W*(*j*, *c*) matrix for that bin. For assessing the robustness this relationship, we defined the **r**elative **w**eight **c**ontribution (RWC) of each profile in a given bin. We estimated the RWC by simply normalizing the weights of W for the bin on the profile with the maximum contribution, which corresponds to $R^{w}_{(j,c)} = W\left( {j,c} \right)/{ \hbox{max} }\left[ {W\left( {j,} \right)} \right];\quad {\text{where }}R^{w}_{(j,c)}$ is the RWC of the profile *c* in the *j*th bin, *W*(*j*, *c*) is the original weight estimate from the W matrix for *c* in that bin and max[*W*(*j*)] is the maximum weight for that bin over all the contributions. We then examined the frequency by which two given chromatin profiles co-occurred in the same bin considering all possible pairs among the profiles for decreasing RWC thresholds (Fig. 6). We found that, by relaxing the weight contribution threshold, specific profile combinations are favored. The most striking association is observed between profiles ActProm and TxInit. At the 95% of the maximum weight, the 3% of the ‘Active Promoter’ bins are also assigned to ‘Transcription Initiation’. This percentage increases up to 17% and then 35% when the RWC threshold decreases to the 75% and the 50% of the maximum contribution, respectively. This trend is quite concordant when we looked at the fraction Transcription Initiation bins that were progressively assigned to ‘Active Promoter,’ which reaches more than 30% at the half of the maximum contribution used in the original assignment, indicating that the two profiles are strictly interconnected. A similar trend is also observed when we looked at the fraction of bins originally assigned to RepChr that were progressively marked as GenBd, which increases up to 35% with the least stringent threshold. Despite this, the relationship between the two profiles seems not symmetric, as indicated by the much smaller fraction of GenBd bins ambiguously assigned to RepChr at the half of the maximum contribution (19%). This behavior suggests that, in the Repressed Chromatin category, a consistent degree of uncertainty in the bin assignment is still present and probably reefers to a less precise identity either in terms of mark composition or spatial organization. This association, however, could be not so surprising as previous studies suggest the presence of histone mark H3K9me3 (the dominant mark of the RepChr profile) in silenced as well as transcribed regions of the genome [41, 42]. A certain degree of association is also found in the Ehn (Enhancer) profile, where the co-assignment with TxInit or ActProm is more recurrent. While the former seems to be moderate (1.3% at RWC = 0.95, 4.3% at RWC = 0.85, 7.6% at RWC = 0.75, 19.6% at RWC = 0.5), the trend is more pronounced in the latter. Indeed, the co-occurrence with ActProm moves from 5% of the total bins marked as Enhancer (RWC = 0.85) up to 10% at RWC = 0.75 and 26% with the lowest cutoff 0.5, indicating that the lines of separation between the two profiles are still not fully delineated.

We then asked how the assignment of additional profiles influences the amount of genomic information captured in each combinatorial pattern. For this purpose, we examined how the mean overlap rate with genomic annotations varies in function of the mean number of profiles assigned per bin (Additional file 1: Figure S5). The mean overlap rate was estimated as the percentage of regions of a given feature covered by a given profile over the total sequences of that feature, averaged across all genomic features significantly enriched in the profile (Fig. 3; Additional file 1: Figure S2). We found that, for some profiles (‘Repressed Regulatory,’ ‘Regulatory Elements’ and ‘Repressed Chromatin’) the degree of overlap constantly increases until all the profiles are assigned, suggesting that the profiles are distributed with a lower degree of clustering with respect to the genomic elements considered. Conversely, profiles ActProm, TxInit, GenBd, Ehn show a flection point between three and four, indicating that assigning at least two other profiles per bin can add further information with an increase of overlap that is, on average, between 7% (‘Enhancer’) and 13% (‘Transcription Initiation’).

### Association between chromatin profiles and gene expression

We decided not to include transcriptomic data from RNA-seq experiments as input signal for the NMF analysis. The RNA-seq expression estimates from the ENCODE consortium database in hESC-h1 were instead used to test whether the different profile distributions are associated with the transcriptional activity and can be functionally interpreted. Specifically, we examined whether the occurrence of specific chromatin profiles over pre-defined gene boundaries correlates with the corresponding expression levels (RPKM). To this aim, we computed the frequency of each chromatin profile over a region of 12 kb (2-kilobases upstream and 10-kilobases downstream) spanning the transcription start site, binned them in increasing ranges of expression levels and identified the most frequent profile in a given bin for a given expression interval. We observed that most of the epigenetic profiles are partitioned in a number of well-delimited regions along the genomic and the expression coordinate, generating diverse bi-dimensional patterns that reflect multiple levels of information about the gene position and the promoter activity (Additional file 1: Figure S6). On a global scale, these bi-dimensional patterns clearly indicate that chromatin profiles can be not only associated with distinct gene elements but are also informative about the transcriptional status of the gene. The frequency distribution for separate chromatin profiles is reported in Additional file 1: Figure S7.

### Chromatin profiles are organized in enrichment patterns that reflect distinct levels of transcription

**Fig. 7:**
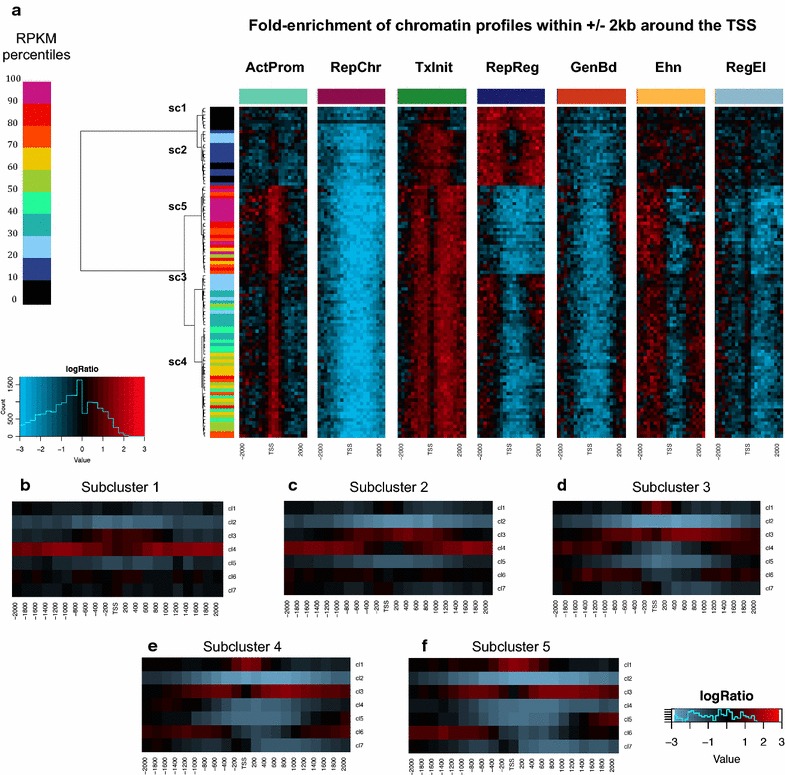
Association between patterns of chromatin profiles and expression levels. **a** Heatmaps showing the hierarchical clustering of sets of genes with similar expression levels on the basis of the chromatin profile frequencies in a region of ±2Kb around the transcription start site. For each profile, frequencies are reported in a separate heatmap, with the corresponding *color*-label positioned on the *top* of each matrix. Each *row* in the matrix corresponds to a specific range of expression and is represented by all genes with an RPKM signal in that interval. Intervals are reported as ranges of percentiles derived from the RPKM distribution. Expression ranges are indicated using *color-scale* labels (on the *left*) from *black* (*lowest*) to violet (*highest*). In each heatmap, a region of ±2Kb around the gene TSS is reported in the *columns* (in 200bp bins). Each cell in the heatmap shows the logarithmic fold-change of the observed frequency over that of the random dataset. *Black cells* indicate a null fold-change (around 1), whilst red and blue reflect positive and negative enrichment, respectively. Unsupervised hierarchical clustering identifies five different sub-clusters that mirror well the different extent of expression. **b**–**f** Average enrichment of profiles in every subcluster. Each cell in a heatmap shows the enrichment of a given profile in a given bin over the 4kb-TSS surrounding region, averaged across all genes in the subcluster. The same *color-scale* as in (**a**) is used

Encouraged by these observations, we asked if the frequency distribution of single profiles could be used to identify enrichment patterns able to discriminate the different ranges of expression. We assigned each gene to a different expression percentile interval on the basis of the corresponding RPKM (reads per kilobase per million of mapped reads). Next, we computed the frequency of each chromatin profile in each expression interval in each bin over a window of ±2 kb around the transcription start site. We next estimated the logarithmic fold-change of the occurrence observed in the real data with respect to the frequency in the random dataset. The heatmap in Fig. 7a clearly shows that, along the genomic and the expression coordinate, chromatin profiles follow different enrichment shapes that resemble well their genomic distribution, consistently with our previous findings (see Fig. 3, 4; Additional file 1: Figure S6). Moreover, hierarchical clustering analysis of the percentile intervals identified a number of clusters and subclusters that accurately reconstruct the full scale of expression. In particular, the clustering analysis allowed us to identify five expression subclusters (Fig. 7a; Additional file 1: Figure S8) specifically associated with a diverse pattern of enrichment (Fig. 7a), thus suggesting a correlation between the occurrence of each profile and the extent of transcriptional activity. Furthermore, expression subclusters are differentially marked by precise sequences of (enriched) profiles around the gene TSS. In particular, five patterns (Fig. 7b–f), discriminate well between the different expression level groups.

### Association between chromatin profiles and transcription factor binding data

**Fig. 8:**
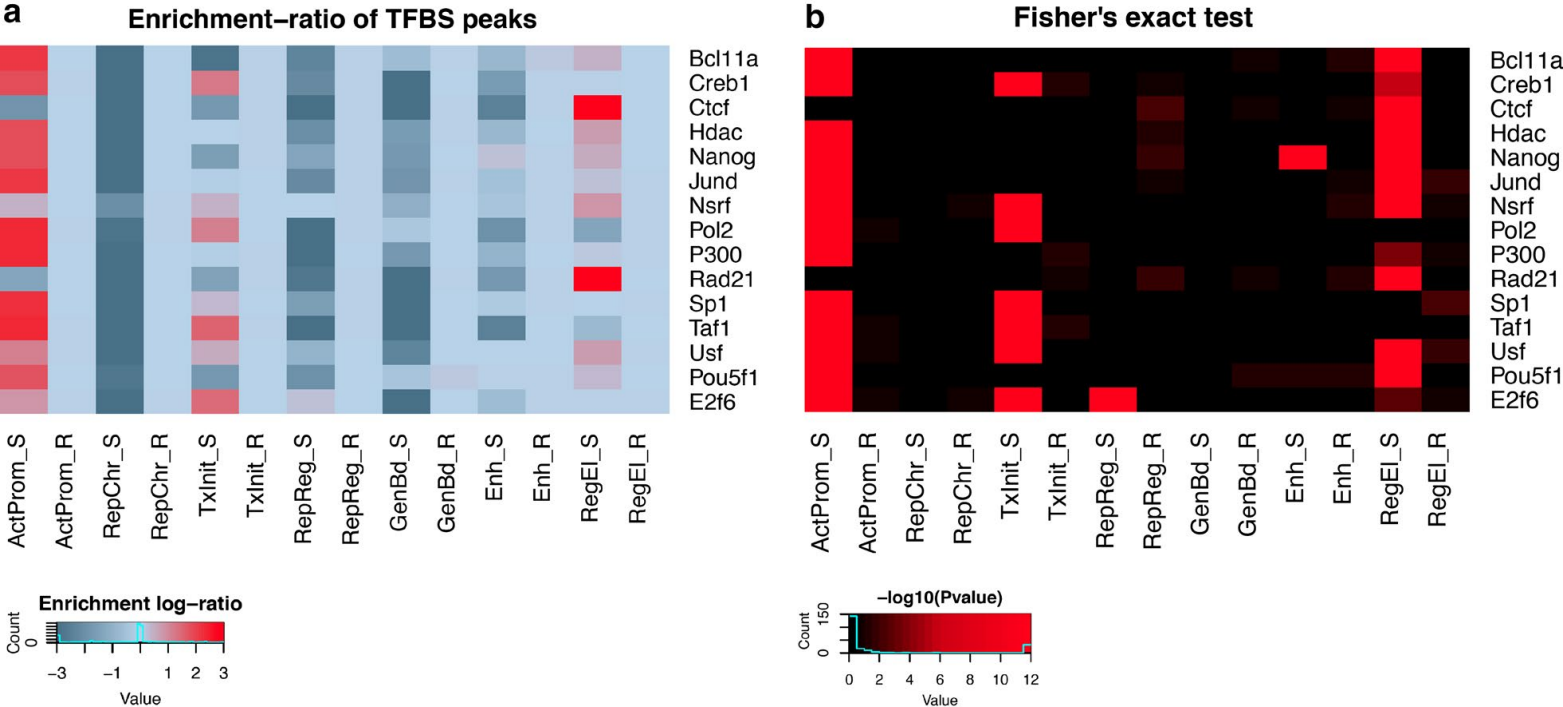
Overlap between chromatin profiles and putative TF binding sites from hESCH1 ChIP-seq data. **a** The heatmap shows the extent of overlap between ChIP-seq peaks from each transcription factor (along *rows*) and the distribution of a given chromatin profile in both the observed and the random data (*columns*). For each possible combination of TF/profile, a fold-enrichment is calculated following the procedure described in “Methods”. **b** Heatmap showing the significance of the enrichment for the same combinations of TFs/profiles represented in (**a**). The *color-scale* indicates the associated *p* value on the basis of the Fisher’s exact test (reported in the −log_10_ form): *black*: *p* > 0.01; *brown* 0.01 > *p* > 0.0001; *dark red* 0.0001 > *p* > 10^−5^; *red p* < 10^−5^

The enrichment of the chromatin profiles relative to ChIP-seq data for a subset of transcription factors allowed us to further investigate the regulatory role of each combination of marks in the control of gene transcription (see “Methods” for details). The overlap analysis shows clear differences in TF enrichment among the profiles (Fig. 8a, b). Unsurprisingly, promoter-associated (profiles ActProm and TxInit) and open chromatin-associated (RegEl) profiles exhibit the strongest patterns of enrichment relative to their random distribution. Among them, chromatin profile ActProm shows clear associations with most of the promoter-activating factors (Bcl11a: f.e. = 9, Fisher’s exact test *p* value = 10^−10^; Creb1: f.e. = 6.45, *p* value = 10^−10^; Nanog: f.e. = 6.1, *p* value = 10^−10^; Jund: f.e. = 8.6, *p* value = 10^−10^; Pol2: f.e. = 10.7, *p* value = 10^−10^; histone acetylase p300: f.e. = 10.64, *p* value = 10^−10^; Sp1: f.e. = 9.24, *p* value = 10^−10^; Taf1: f.e = 10.3, *p* value = 10^−10^; Usf: f.e. = 3, *p* value = 10^−10^; Pou5f1: f.e. = 5.5, *p* value = 10^−10^). Notably, this profile shows significant overlap with the human histone deacetylase 1 protein (Hdac: f.e. = 6, *p* value = 10^−10^), suggesting a possible additional role in the control of the chromatin organization for this factor. The ‘Transcription Initiation’ profile shows the highest enrichment in the cAMP-responsive element binding protein (Creb1: f.e. = 3.46, *p* value = 10^−10^) and for members of the basal transcription machinery (Pol2: f.e = 3.3, *p* value = 10^−8^; Taf1: f.e = 4.8, *p* value = 10^−9^) but with a less pronounced overlap compared to that of profiles ActProm and RegEl. Interestingly, the TxInit profile has relatively stronger enrichment in the E2f6 transcription factor (f.e. = 4.24, *p* value = 10^−8^) compared to profile ActProm. E2f6 is a well-known inhibitor of the E2f-dependent transcription and may negatively regulate RNA polymerase II-dependent promoters via the recruitment of a chromatin remodeling complexes. This result seems to be in agreement with the fact that profile TxInit is found as predominant in the TSS-spanning regions of lowly expressed genes (Fig. 7a, c), supporting the assumption of its bivalent role in the control of the chromatin dynamics. In the same manner, profile RegEl shows relatively higher enrichment for several TFs (Fig. 8b) (Bcl11a: f.e. = 1.6, *p* value = 10^−5^; Hdac: f.e. = 2.14, *p* value = 10^−6^; Nanog: f.e. = 1.81, *p* value = 10^−5^; Nsrf: f.e. = 2.4, *p* value = 10^−5^; p300: f.e. = 1.12, *p* value = 10^−4^; Usf: f.e. = 2, *p* value = 10^−5^; Pou5f1: f.e. = 1.45, *p* value = 10^−15^) but with lower levels of overlap than found in promoter-associated profiles (ActProm and TxInit). In contrast, the RegEl profile is found to be enriched in the CCCTC binding insulator protein (CTCF: f.e. = 116, *p* value = 10^−12^) and the double-strand break repair Rad21 homolog protein (Rad21: f.e. = 121, *p* value = 10^−12^), another chromatin binding protein known to cooperate with the CTCF-mediated insulator complex in modulating enhancer–promoter interactions [43].

### Comparison with different chromatin segmentation approaches

We compared our chromatin profile distribution with that of ChromHMM (9), which is a common chromatin segmentation technique. We did not compare our method with the Segway algorithm [10] because its 1-bp segmentation resolution would not permit to easily compare the different profile/state distributions.

Both ChromHMM and EpicSeg have been shown to be effective when used close to 13 chromatin states [11, 44]. Therefore, an additional NMF model with a factorization rank of 13 was also tested for the comparison. This is also the maximum number of states we can compute since it corresponds to the number of epigenetic marks in the input data matrix.

We assessed the ability of the methods to recover functional information about biologically relevant regions and to correctly predict the presence of distinct functional elements in terms of their sensitivity (true positive rate), specificity (true negative rate), precision (number of true positives divided by the sum of true positives and false positives) and accuracy (total fraction of correct predictions).

The heatmaps in Additional file 1: Figure S9 show the enrichment of the epigenetic profiles (states) generated by NMF with r = 7 (denoted as NMF^7^), NMF with *r* = 13 (NMF^13^), the ChromHMM 7-states model (ChromHMM^7^) and the ChromHMM 13-states model (ChromHMM^13^). The enrichment patterns of NMF^7^ are very similar to those of ChromHMM^7^ and ChromHMM^13^, while the NMF^13^ model is much less effective, confirming that a level of factorization of 7 is sufficient for the NMF model to capture the combinatorial information contained in the chromatin profiles.

**Fig. 9:**
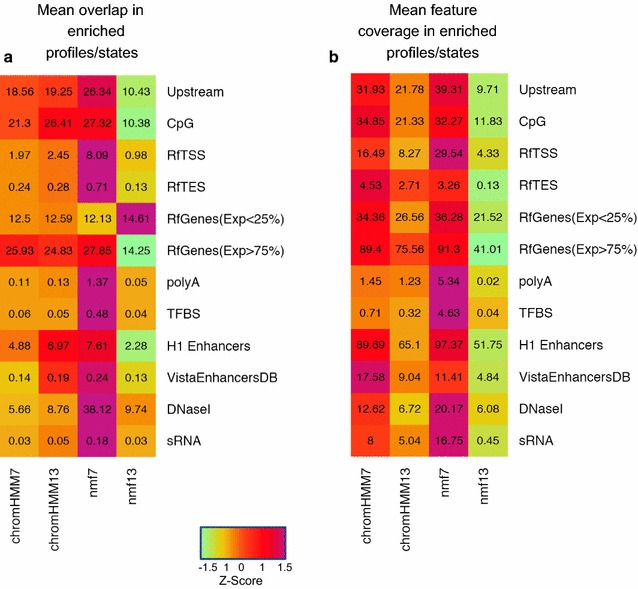
Overlap and coverage levels of different genomic elements using enriched profiles/states in each method. Chromatin segmentation approaches are reported in columns, genomic features in rows. Each cell in the heatmap indicates the amount of overlap (**a**) or coverage (**b**) observed intersecting a feature with any profile/state specifically enriched in that feature using NMF or ChromHMM-based methods. The extent of overlap is represented as the mean percentage by which an enriched profile/state overlaps the feature (**a**). Similarly, we represent the coverage as the mean percentage of a given feature covered by any enriched profile/state. The *color-scale* (from *green* to *purple*) mirrors the amount of information retrieved for each pair of feature/method. Genomic features labels indicate: Upstream = 1kb upstream region from the Refseq TSS; CpG = CpG islands; RfTSS = genomic window of ±50bp (“Methods”) around the Refseq TSS; RfTES = genomic window of ±50bp around the Refseq TES; RfGenes(exp <25%) = Refseq genes with mean RPKM value smaller than the 25th percentile; RfGenes (exp >75%) = Refseq genes with mean RPKM value higher than the 75th percentile; PolyA = polyAdenylation-sites from PolyA-database; TFBS = conserved transcription factor binding sites; H1-enhancers = super enhancers regions precited in hESC from the dbSuper database; vistaEnhancers = experimentally validated enhancer regions in human; DNaseI = DNase hyper-sensitive sites from ENCODE project database; sRNA = predicted small RNAs

Figure 9 illustrates the ability of each method to retrieve annotated features. We restricted the analysis to all the combinatorial profiles (states) that were significantly enriched in a given feature, and used the enriched profiles (states) to determine the degree of genomic overlap. Specifically, the amount of information retrieved was measured using two different indicators: (i) the mean profile/state overlap and (ii) the mean feature coverage. The mean profile overlap was computed as the fraction of bins of a profile P overlapping at least one element of the feature G, averaged across all profiles/states enriched in that feature. The mean feature coverage was estimated as the fraction of elements in G matching the profile P, averaged across all profiles/states found to be significantly enriched in that feature. We observed that the two indicators gave similar results when we looked at the performance between the different techniques (Fig. 9a, b). Compared to our NMF model and ChromHMM approaches, the NMF^13^ model shows very weak overlap in all the features examined (Fig. 9a), with the exception of low expressed Refseq genes (mean profile overlap = 14.6%). A significant drop of overlap from NMF^7^ to NMF^13^ is also observed in terms of mean feature coverage (Fig. 9b), which is in accordance with our previous results.

Interestingly, we noted that our NMF^7^ model slightly outperforms ChromHMM^7^ and ChromHMM^13^ both in terms of profile/state overlap and feature coverage for almost all the features considered (Fig. 9a, b). Indeed, the NMF^7^ model exhibits the highest overlap rate in 11 out of 12 features (91%). Remarkably, the NMF^7^ approach and the ChromHMM^7^ tend to perform better than the ChromHMM^13^ model in terms of feature coverage. In 9 out of 12 features (75%), our NMF model shows the best mean coverage, confirming the ability of the NMF profiles to retrieve a larger fraction of biological information from distinct types of functional regions in the genome. In both analyses, the most striking differences are observed for Refseq transcription start sites (mean overlap: NMF^7^ : 8%, ChromHMM^7^ : 1.97%, ChromHMM^13^ : 2.45%; mean coverage: NMF^7^ : 23.5%, ChromHMM^7^ : 16.4%, ChromHMM^13^ : 8.2%), upstream regions (mean overlap: NMF^7^ : 26.3%, ChromHMM^7^ : 18.5%, ChromHMM^13^ : 19.2%; mean coverage: NMF^7^ : 39.3%, ChromHMM^7^ : 31.9%, ChromHMM^13^ : 21.8%), smallRNAs (mean overlap: NMF^7^ : 0.18%, ChromHMM^7^ : 0.03%, ChromHMM^13^ : 0.05%; mean coverage: NMF^7^ :16.7%, ChromHMM^7^ : 8%, ChromHMM^13^ : 5%) and DNase hypersensitive sites (mean overlap: NMF^7^ : 38.1%, ChromHMM^7^ : 5.6%, ChromHMM^13^ : 8.7%; mean coverage: NMF^7^ : 20.1%, ChromHMM^7^ : 12.6%, ChromHMM^13^ = 6.7%).

To assess the generality of these observations, we repeated the two analyses illustrated above varying the set of enriched profiles/states used for each method. We considered all possible combinations of size L ≤ N among the profiles/states, where N corresponds to the total number of enriched profiles/states detected by a given method in a given feature (Additional file 1: Table S1). The distribution of the mean profile overlap and the mean feature coverage generated for each feature are shown in Additional file 1: Figure S10. We found that, in almost all cases, the NMF^7^ approach gives similar or better overlap compared to both the ChromHMM^7^ and the ChromHMM^13^ model, suggesting that this trend is unlikely to depend on the selection of a specific combination of enriched profiles (Additional file 1: Figure S10a, b).

**Fig. 10:**
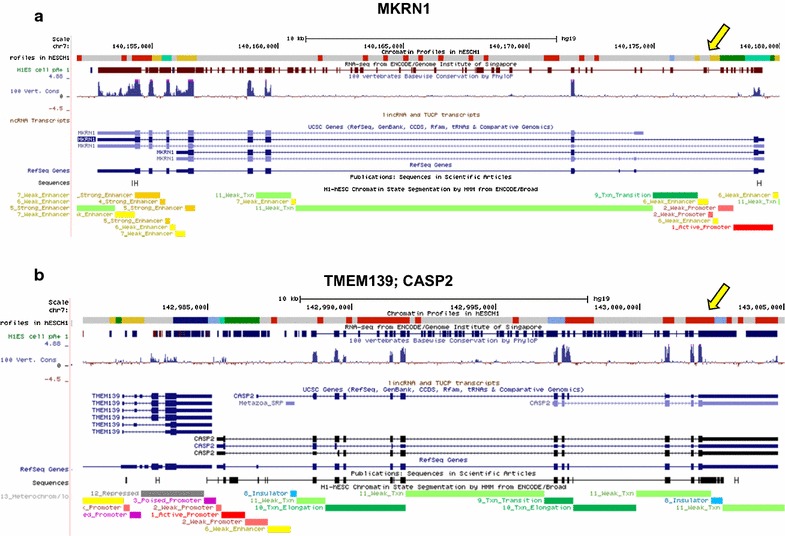
Examples of genomic visualization of the NMF-based epigenetic profiles on the UCSC Genome Browser. Chromatin profiles are compared with the UCSC Refseq gene annotation tracks at the TMEM139/CASP2 and the MKRN1 loci. NMF-profiles are highlighted with the same color scheme adopted in this work and are displayed in the first track of the panel, as indicated by the yellow arrow on the right. **a** Genomic visualization of chromatin profiles over a 25Kb-region encompassing two different genomic loci: the TMEM139 (transmembrane protein 139) and the CASP2 gene. A specific chromatin transition ‘ActProm > TxInit’ is found exactly on the TSS of the CASP2 gene, suggesting the presence of a functionally active promoter. Chromatin profile GenBd is also detected multiple times on both intronic and exonic regions of the gene, indicating that the CASP2 is transcriptionally active in hESC-H1 cells. The NMF-approach also identified a repressive chromatin region (profile RepReg) on the 3′ end and a potential enhancer element over the 5′ end of the TMEM139 gene, that are also confirmed by ChromHMM predictions in the bottom track. **b** Chromatin profiles at the MKRN1 locus (the Makorin ring finger protein1). MKRN1 appears to be well-expressed in hESC-H1 cells as indicated by the ‘TxInit > ActProm > TxInit’ chromatin motif over the TSS region and the Gene Body Transcription profile that frequently appears in both introns and the last exons of the gene. On the left of the figure, a putative active enhancer (i.e. the chromatin profile sequence ‘Enh > ActProm > Enh’) is predicted over the 3′ end of the gene. This prediction appears to be concordant with the ChromHMM annotation, as indicated by the ‘strong-enhancer’ label in the corresponding chromatin segmentation track. Finally, a putative CTCF-binding region (profile RegEl) also appears in the first intron, suggesting a functional role in the control of MKRN1

Next, we tested the performance of the different approach in the prediction of some important functional genomic features (TSSs, upstream regions, and putative H1 enhancers) using, for each model, the full set of enriched profiles/states. The true positive rate was estimated considering all the bins assigned to any of the enriched profile/state and having (or not) a minimum overlap (1 bp) with the analyzed feature. A number of intragenic (or intergenic) intervals lacking any annotation for the specific feature were assumed as negative controls for the test. We found that, when all the enriched profiles/states were used, our approach has in general the same predictive power of the ChromHMM models (Additional file 1: Figure S11a–c). We also observed that, in the prediction of the Refseq TSSs (Additional file 1: Figure S11a), the NMF^7^ model shows a marginal drop in sensitivity and a small gain in precision and specificity compared to ChromHMM-based approaches. As an example, a UCSC Genome Browser representation of the chromatin profile distribution generated by the NMF^7^ model at level of two distinct genomic regions on the human chromosome 7 (the TMEM139/CASP2 genomic loci and the MKRN1 locus) is reported in Fig. 10.

### Chromatin profiles distribution in IMR90 ENCODE data

We also tested the NMF approach on a different human dataset, i.e., IMR90 (fetal lung fibroblasts), another cell line well characterized by a large number of functional chromatin assays publicly available on the ENCODE catalog [5]. We compared the results on the two datasets using both an IMR90-derived coefficient matrix (H) and that derived from the hESCs-H1 cell line. A Pearson correlation analyses was performed using all possible pairwise comparison between the profiles (Additional file 1: Figure S12a, b). We found that, in both cases, most of the IMR90 profiles are well correlated with the corresponding hESC profile (as suggested by the red diagonal in the Additional file 1: Figure S12a, b), indicating that the two datasets tend to be similar in terms of genomic organization and chromatin mark composition. The only major difference is observed for the profile RepReg. In hESCs, this epigenetic profile is mainly dominated by the H3K27me3 histone modification with almost no traces of other mark contributions (Fig. 2a). Conversely, the IMR90 profile s4 is described by moderate H3K79me1 and CTCF mark contributions (data not shown) and partially correlates with the hESC profiles GenBd and RegEl. In contrast, we found that hESC RepReg profile appears closer to the IMR90 profile ‘s2,’ which is represented, indeed, by a combination of the two repressive marks H3K27me3 and H3K9me3.

## Discussion

In this work, we demonstrated the usefulness of the non-negative matrix factorization for the systematic characterization of the chromatin functions. This is an unsupervised classification technique that uses a signal decomposition algorithm to reduce the dimensionality of multivariate datasets to a restricted number of combinatorial components [14, 45, 46]. The ability of NMF to use the sparse data makes the approach particularly appropriate for the analysis of NGS experimental data. We show here that the method can discover functional relationships among different epigenetic marks and permits to extract a number of combinatorial profiles useful for the biological interpretation of the broad spectrum of the chromatin functions.

As discussed in previous studies, the most critical parameter in common chromatin segmentation techniques is represented by the expected number of chromatin profiles (states) chosen to capture meaningful combinations of marks. One of the main advantages of the NMF approach is that the number of chromatin profiles is not arbitrarily set. Indeed, here we statistically assessed the stability of the data for an increasing number of profiles and selected the value at which the clustered data started being significantly stable compared to the random profile distribution. This approach allowed us to identify seven different chromatin profiles that better represents the most recurrent combination of marks.

We tested NMF on a combined epigenetic dataset of 13 chromatin marks, encompassing 9 histone modifications, one histone variant, two transcription factors binding data (Pol2, CTCF) and one chromatin accessibility mark (DNase hypersensitive site assay), previously mapped in hESC and IMR90 lines. A complete list of the analyzed datasets is reported in the Additional file 2. We found that epigenetic profiles are composed by different chromatin patterns that resemble quite well the functional diversity of single marks, highlighting the ability of NMF in capturing spatial relationships among different epigenetic signals. When compared to a number of well-annotated features and other functional elements, we also found that combinatorial profiles are predominantly associated with specific genomic contexts, which suggests the usefulness of the NMF approach in extracting biologically interpretable information from meaningful combinations of marks.

We also found that chromatin profiles are more effective than single marks in recovering known functional elements and observed that most profiles are distributed following specific bi-dimensional patterns along the genomic and the expression coordinates. This strongly suggests that chromatin profiles are not only related to the genomic localization of distinct functional elements but can also be correlated to the level of transcriptional activity. Specifically, we showed that, around the promoter region, chromatin profiles are organized in signatures that preserve positional information and are able to mirror well the different ranges of expression. Our data suggest the presence of an almost symmetric mechanism of chromatin activation, which could propagates progressively from the gene TSS toward distal upstream and downstream regions through a defined number functional chromatin states (Fig. 7b–f). When compared to those obtained from ChromHMM, our combinatorial profiles share similar patterns of enrichment in terms of genomic organization, but tend to have better sequence overlap with a variety of functional elements.

We also used the enriched profiles from both methods to verify how well they can predict annotated regions in the genome and showed that the NMF model has very similar predictive power, but has a slightly higher precision and specificity in the prediction of Refseq TSSs, with a very small drop in terms of sensitivity.

Furthermore, our results are not specific for the selected cell line (hESC-H1) since the profiles obtained using another one (IMR90) provides very similar results.

Despite these observations, there are still a number of limiting factors that have to be taken into account and that will require additional efforts to further improve the accuracy of the approach. First of all, we analyzed how the chromatin profile assignment varies as function of the single profile contribution and we found that specific profile combinations are more recurrent than others, suggesting that there is still a fraction of uncertainty in determining the correct chromatin status for specific groups of bins. We also found that, in some cases, assigning more than one profile makes the chromatin segmentation procedure even more informative, suggesting that some genomic information could be missed in the bin assignment. In this context, more statistically rigorous approaches to redefine bin/profile relationships will likely be needed. Moreover, further methodological improvements will be required to optimize the signal decomposition algorithm in the integrative analysis of multiple epigenetic datasets. Although the technique has been shown to be effective in capturing most important functional mark interactions, the detection of combinatorial information for some histone mark distributions (as for H3K27me3, H3K9me3 and H3K4me1) still remains a challenging task. This consideration arises from the fact that such histone modifications tend to be compressed into a unique chromatin profile (as for RepReg, RepChr, Ehn) rather than gradually fluctuate over different functional states, making the approach less sensitive to local patterns of mark interactions. An example of such patterns is particularly evident in undifferentiated cells, where distinct bivalent domains of chromatin modifications (e.g., H3K27me3/H3K4me3 and H3K4me3/H3K9me3) were found at poised promoters of lineage-specific genes [47, 48]. This limitation, however, seems not to be strictly related to the NMF approach as a similar trend also arises with previously proposed chromatin segmentation techniques [9–11, 49], thus indicating that a certain degree of complexity still persists in the interpretation of this kind of data

Another common limiting factor is represented by the large amount of memory and time resources required to process such large amount of epigenetic signals, which often makes the analysis not very practical. For the hESC dataset, both the NMF and the ChromHMM performed in the same time range on a Intel^®^Xeon^®^ 56 Gb RAM multi-core machine. It is, however, worth mentioning that the implementation of the NMF technique in the R environment allows easier integration of statistical function and appropriate libraries normally used in downstream analyses. We believe that this technique can improve the limited repertoire of tools and algorithms currently available in the analysis of high-dimensional epigenetic datasets, thus facilitating the development of novel frameworks for a more accurate characterization of the chromatin activity (the implemented pipeline is provided in Additional file 3).

## Conclusion

In our work, we employed an unsupervised learning technique able to integrate multiple types of chromatin data and capture meaningful combinations of signals that can be used to systematically classify the functional states of the epigenome. We identified seven combinatorial profiles that show distinct genomic distributions and strongly correlate with well-annotated features in the genome. Moreover, we found that, around the gene TSS, most of the profiles are organized in specific enrichment patterns that mirror the degree of transcriptional activation, highlighting the ability of the NMF approach in recovering biologically relevant information from multiple ChIP-seq experiments. Clearly, there is room for improvement in the integrative analysis of multiple epigenetic datasets. We chose a representative set of 13 different epigenetic marks among the most abundant and well-characterized histone modifications available in public databases. However, the number of histone modifications described in the public domains keeps increasing and the NMF approach can and should be extended to additional types of marks and other epigenetic signals. A higher resolution of the chromatin segmentation step could also be further explored although this would lead to a very memory intensive procedure and will most likely require a further development of the software in terms of computational efficiency.

## Additional files

**Additional file 1.** Supplementary figures and tables with legends (in PDF format). **Figure S1**: Selection of the best factorization rank. (**a**) The plot shows the variation of the cophenetic correlation coefficient (on the Y-axis) for both the real (blue) and the random (red) data for increasing values of *r*. Each point in the real data is obtained after 30 runs of a single NMF analysis using the factorization rank indicated on the X-axis. For the random data, each point indicates the mean cophenetic coefficient obtained by repeating the NMF analysis 30 times at a given factorization rank. The cophenetic coefficient in the real dataset becomes stable at *r* = 7 remaining at about 0.99 up to *r* = 11, whilst the stability of the random dataset dramatically drops in the same interval. Within this range, the cophenetic coefficient obtained in the real dataset is more than 4-fold the standard deviation of the coefficients in the random data. **b** The plot shows the trend of the sparseness in the W (basis) and H (coefficient) matrices over the same range of factorization ranks, in both the real and the random dataset. **Figure S2**: Significance of chromatin profile enrichment in distinct genomic features. The heatmap shows for each epigenetic profile, the significance of the enrichment compared to different types of genomic features and functional regions of the genome. The significance of the enrichment is assessed using a Fisher’s exact test with a *p* value of 10^−3^ as statistical threshold. The color-scale from blue to red indicates the significance of the test as follows: blue: *p* value > 0.01; purple: 0.01 > *p* value > 0.001; dark red: 0.001 > *p* value > 10^−5^; red: *p* value < 10^−5^. A specific biological label is assigned to each profile in order to facilitate its biological interpretation on the basis of the enrichment observed (top-bottom): ActProm = Active Promoter (profile 1); TxInit = Transcription Initiation (profile 3); RepReg = Repressed Regulatory Regions (profile 4); Ehn = Enhancer Regions (profile 6); RegEl = Regulatory Elements (profile 7); GenBd = Gene Body Transcription (profile 5); RepChr = Repressed Chromatin (profile 2). Genomic features are the same represented in Figure 3: CAGE = hESC-H1 CAGE clusters from ENCODE; RfTSS = Refseq Transcription Start Sites; RfTES = Refseq Transcription End Sites; 5UTR = Refseq 5’untranslated region; 3UTR = Refseq 3’unstranslated regions; H1 Enhancers = Superenhancer regions from hESC; CpG = CpG islands; Upstream = 1Kb upstream regions from Refseq TSSs; DNase1 = hESC DNase1 Hypersensitive sites from ENCODE; TFBS = Conserved transcription factor binding sites from the Transfac Matrix Database; 5C = Chromatin conformation capture carbon copy data from hESC; EnhancersDB = experimentally validated enhancer elements from the VistaEnhancer Dabatabse; Rf = Refseq genes; Int = intronic sequences from Refseq genes; Ex = exonic sequences from Refseq genes; PolyA = predicted poly-adenylation sites; sRNA = small RNAs; HMMhetero = predicted heterochromatin regions in hESC. **Figure S3**: Frequency of transition between epigenetic profiles. The grid shows the occurrence of each transition for all possible pair-wise combination of profiles. Each cell in the heatmap represents the enrichment of a transition A → B from the profile indicated in each row (A) to the profile reported in the corresponding column (B). Only transitions between consecutive profiles (i.e. regions of the profiles not separated by one or more unassigned bins) are considered. For each combination, the enrichment is calculated as the logarithm of f_AB_(s)/f_AB_(r) where f_AB_(s) is the fraction of the regions of A followed by any region of B over the total of A-regions observed in the real sample and f_AB_(r) the fraction of A-regions followed by any region of B over the total number of A-regions in the random dataset. Chromatin profiles are indicated using the same labels as in Figure 3 and Supplementary Figure 2. **Figure S4**: Comparison between the recovery of poly-adenilation sites in chromatin profiles and single epigenetic marks. The plots show the Receiver Operating Characteristic (ROC) curve generated to compare the performance of different chromatin profiles with those of single marks for the recovery of known poly-adenilation sites. The curve is generated by measuring the TPR (true positive rate) and FPR (false positive rate) at increasing prediction thresholds according to the fraction of annotated poly-A sites covered by all bins having a signal above that threshold. Each epigenetic mark is evaluated on the basis of the sigmoid-transformed normalized coverage track, as reported in the input matrix V_j,k_ of the NMF. Each chromatin profile is quantitatively evaluated using the weight distribution over all genomic intervals (the columns in the W_j,c_ matrix). To provide a better visualization of the results only the most representative set of chromatin profiles and marks are represented for each feature. **Figure S5**: Recovery of genomic information using ambiguous profile assignment. The plot gives a representation of how the genomic overlap changes in function of the number of different profiles assigned to a bin using their relative weights sorted in decreasing order (i.e. the values of the W-matrix). The amount of genomic information retrieved is reported on the Y-axis as the mean rate of overlap considering all genomic features significantly enriched in a given profile. Each chromatin profile is denoted with the same label and color scheme previously adopted in the main text. **Figure S6**: Chromatin profile assignment according to genomic position and gene expression. The color-code heatmap is used to represent chromatin profile assignment over a 12Kb region (2Kb upstream and a 10Kb downstream) around the TSS in a subset of 1000 genes from GENCODE (GRCh37)-database binned in 200bp consecutive genomic intervals. Genes are sorted in decreasing order according to the RPKM expression vaule and reported on the Y-axis. The X-axis indicates the genomic distance from the GENCODE Transcription Start Site, which is positioned at zero. Each profile is indicated using the same color legend previously adopted in this work: ActProm (Active Promoter) = light green, RepChr (Repressed Chromatin) = purple, TxInit (Transcription Initiation) = dark green, RepReg(Repressed Regulatory) = blue, GenBd (Gene Body Transcription) = red, Enh (Enhancer Regions) = yellow, RegEl (Regulatory DNA Elements) = grey. The white vertical line on the left side of the heatmap shows the exact TSS position. The fraction of genes with length corresponding to each interval from the TSS is reported in the upper panel. The yellow bar at the bottom of the graph represents genes with length greater than 10 Kb (more than the 70% of the total number of genes). **Figure S7**: Frequency of chromatin profiles according to expression and distance from the gene TSS. Each plot shows the distribution of a specific epigenetic profile in a bi-dimensional space defined by TSS-surrounding region and the level of gene expression (RPKM). A 12Kb region (2Kb upstream the TSS and 10Kb downstream) is represented on the X-axis as a sequence of 200 bp genomic intervals. Each column along the axis is labeled with its distance from the TSS (x = 0). On the Y-axis, RPKM expression estimates are reported as percentiles in decreasing order from the top (highest expression) to the bottom (lowest range of expression). Each cell in the matrix shows the frequency by which a given profile occurs in a given interval from the TSS and a given range of expression. We compute this frequency as c^i^(x,y)/c^i^tot , where c^i^(x,y) is the number of bins of profile i observed in the genomic interval x and the expression range y and c^i^tot is the total number of genomic bins belonging to the profile. The ‘Repressed chromatin’ (RepChr) profile is omitted in this figure since no distinct patterns were observed on the basis of the TSS-distance and the expression. **Figure S8**: Distribution of gene expression in different subclusters. Each box-plot in the graph shows the expression distribution from all genes contained in each of the subclusters represented in Figure 7. Sub-clusters are numbered from 1 to 5 according to their median expression value. Gene expression levels are reported in RPKM (reads per Kilobase per Million of Mapped Reads) on the Y-axis. **Figure S9**: Feature enrichment distribution across profiles/states using different methods. The enrichment distribution (reported as Fisher-test Odd-ratio) of different genomic features for each profile identified in our study is displayed as color-scale heatmap (**a** top-left) and compared with that obtained from different chromatin segmentation approaches: (**b** top-right) NMF with factorization rank = 13; (**c** bottom-left) ChromHMM with 7 states; (**d** bottom-right) ChromHMM with 13 states. In heatmaps from **b** to **d**, profiles/states are numbered in a completely independent manner since there are no relationships between numerical orders in the different plots. Genomic features indicated in the columns are those reported in Fig. 3. CAGE = hESC-H1 CAGE clusters from ENCODE; RfTSS = Refseq Transcription Start Sites; RfTES = Refseq Transcription End Sites; 5UTR = Refseq 5’untranslated region; 3UTR = Refseq 3’unstranslated regions; H1 Enhancers = Superenhancer regions from hESC; CpG = CpG islands; Upstream = 1Kb upstream regions from Refseq TSSs; DNase1 = hESC DNase1 Hypersensitive sites from ENCODE; TFBS = Conserved transcription factor binding sites from the Transfac Matrix Database; 5C = Chromatin conformation capture carbon copy data from hESC; EnhancersDB = experimentally validated enhancer elements from the VistaEnhancer Dabatabse; Rf = Refseq genes; Int = intronic sequences from Refseq genes; Ex = exonic sequences from Refseq genes; PolyA = predicted poly-adenylation sites; sRNA = small RNAs; HMMhetero = predicted heterochromatin regions in hESC. **Figure S10**: Overlap and coverage of functional genomic regions with different combinations of enriched profiles/states. Each boxplot panel shows the distribution of the mean overlap over all possible combinations of profiles/states in each feature (**a**) and the mean coverage of a given feature across profile/state combinations (**b**) using different approaches. Data are represented such that any point in a boxplot indicates the mean overlap (or coverage) of the represented feature observed for a single combination of profiles/states. The overlap/coverage distributions are highlighted with different colors according to the tested method: ChromHMM/13states = green; ChromHMM/7states = darkblue; NMF/13profiles = yellow; NMF/7profiles = orange. **Table S1**: Combinations of enriched profiles/states used to test the overlap/coverage in a set of genomic features. The table shows for each genomic feature (first column) the list of profiles (states) enriched in that feature and the number of all their possible combinations with size L ≤ N (where N is the number of enriched profiles/states for that feature) obtained by each method. Distributions of the mean overlap/coverage for such profiles/states combinations are reported in Supplementary Fig. 10. Feature labels reported in the most-left column are (top-bottom): 1 Kb-upstream = 1 Kb upstream regions from Refseq TSS; CpG Islands; 100 bp window surrounding Refseq Transcription Start Site; 100 bp windows surrounding Refseq Transcription End Site; Refseq genes over the 75th percentile of the RPKM expression distribution; Refseq genes under the 25th percentile of their RPKM expression distribution; PolyAdenylation sites; Conserved transcription factor binding sites; Superenhancer regions from hESC; Experimentally validated enhancer elements from the VistaEnhancer Dabatabse; hESC DNase1 Hypersensitive sites from ENCODE; small RNAs; Predicted hESC heterochromatin regions from ChromHMM. **Figure S11**. NMF and ChromHMM performance in the prediction of distinct functional elements in the genome. Each graph compares the performance of the NMF-based approach with that of ChromHMM (with 7 and 13 states) for the prediction of functionally relevant regions such as Refseq transcription start sites (**a**), hESCH1-super enhancers (**b**) and Refseq 1Kb-upstream regions (**c**). For a given feature, only significantly enriched profiles/states identified by each method are used as predictors. True positives are bins assigned to an enriched profile/state and having a minimum overlap (1 bp) with the considered feature. Negative controls are provided as a list of intragenic (or intergenic) intervals without any evidence of annotation for that feature. **Figure S12**. Comparison of the NMF chromatin segmentation approach in different cell lines. The two heatmaps show Pearson correlation index distribution between chromatin profiles from hESCH1 and IMR90 cell lines using all possible pairwise comparisons. The two cell lines were correlated using either single chromatin mark coefficients from the H-matrix (**a**) or genomic enrichment distribution (**b**) of single profiles in a number of genomic features (the same reported in Fig. 3 and Supplementary Fig. 9a, b, c, d). Epigenetic profiles detected IMR90 (columns) are labeled with an ‘s’ followed by numeric assignment (1–7). hESC chromatin profiles are reported in rows using the same label system adopted in the previous figures.

**Additional file 2.** List of samples collected and analyzed in this study (Excel format). For each sample, the table reports: (1) the cell type, (2) the genetic information analyzed through the genomic assay, (3) the epigenetic mark, (4) the sample identifier from GEO, (5) the name of the BAM/BED mapping file, (6) the type of the alignment data (processed or not), the number of the biological replicate from the same lab/experiment, (8) the name of the structure which generated the experiment, (9) the link to the sample file. All ChIP-seq/DNase-seq alignment files were downloaded from the Gene Expression Omnibus database and were provided as part of the NIH Roadmap Epigenomics project and the ENCODE project database.

**Additional file 3.** Archive file (ZIP format) containing all the scripts of the computational pipeline implemented in our work (FGandolfi_pipeline.zip).

## Abbreviations

TSS: transcription start site
CAGE: cap analysis of gene expression
TFBS: transcription factor binding site
Pol II: RNA polymerase II
CTCF: CCCTC binding factor
GEO: Gene Expression Omnibus database
p(1-7): chromatin profiles 1-7
f.e: fold-enrichment
RPKM: reads per kilobase per million of reads mapped
HMM: Hidden Markov model
NMF: non-negative matrix factorization
ChIP-seq: chromatin immunoprecipitation and next-generation sequencing
IDR: irreproducible discovery rate
ROC: receiver operating characteristic
ActProm: Active Promoter
RepChr: Repressed Chromatin
TxInit: Transcription Initiation
RepReg: Repressed Regulatory
GenBd: Gene Body Transcription
Ehn: Enhancer Regions
RegEl: Gene Regulatory Elements
