# Supplementary material for: A computational approach for the functional classification of the epigenome
Source: Epigenetics Chromatin. 2017 May 15;10:26. doi: 10.1186/s13072-017-0131-7 (PMC5433140; doi:10.1186/s13072-017-0131-7)

# Additional File 1

## Supplementary data to

# "A computational approach for the functional classification of the epigenome"

Francesco Gandolfi[1], Anna Tramontano [1,2]

[1]Department of Physics, Sapienza Universita' di Roma, P.le Aldo Moro 00183 Rome, Italy

[2]Istituto Pasteur, Fondazione Cenci Bolognetti, Viale Regina Elena 291, 00161, Rome, Italy

# Figure S1 – Selection of the best factorization rank

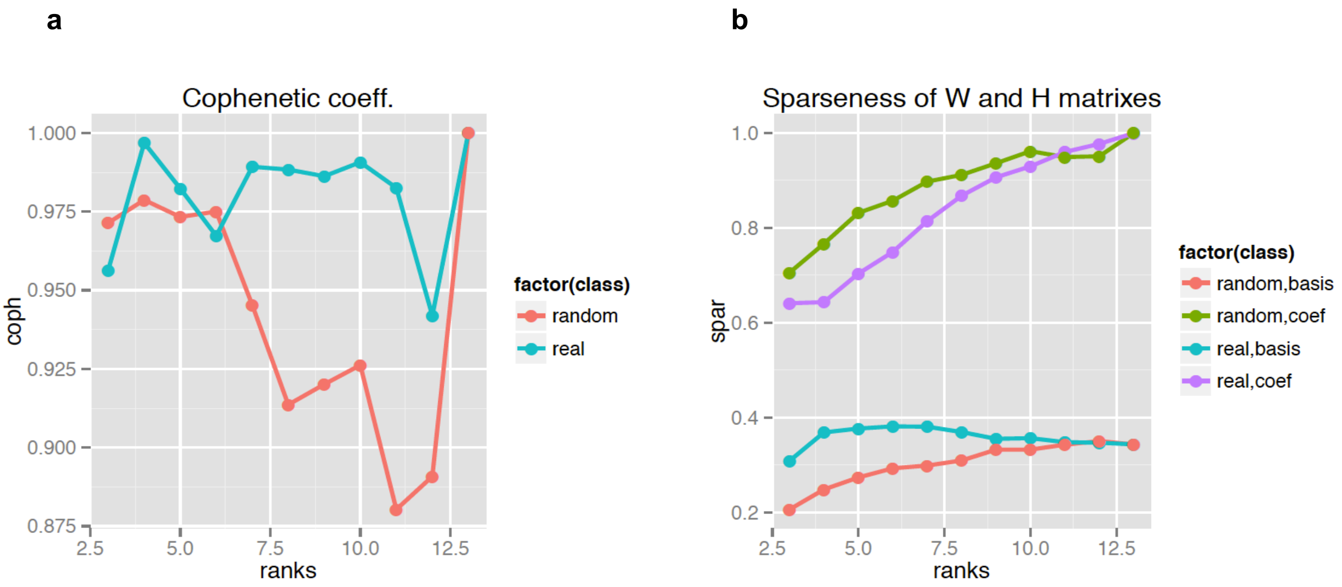

**Figure S2 – Significance of chromatin profile enrichment in distinct genomic features**

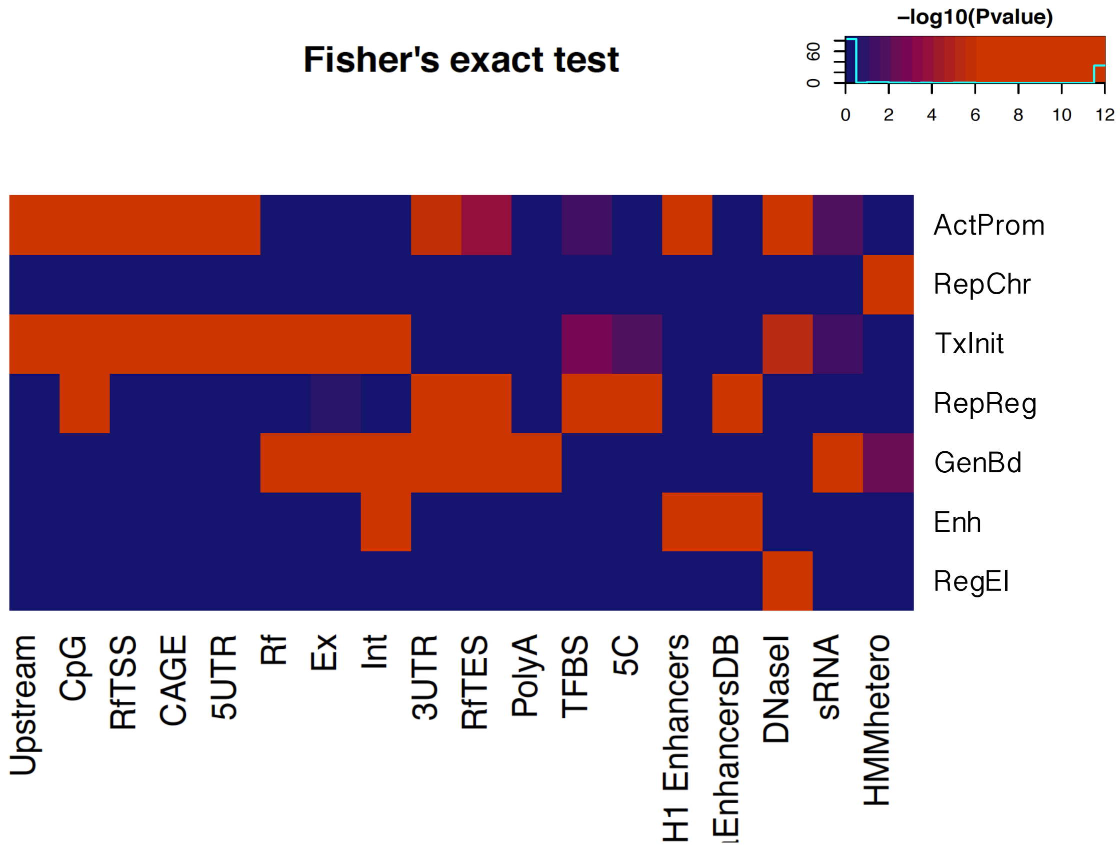

## Figure S3 – Frequency of transition between epigenetic profiles

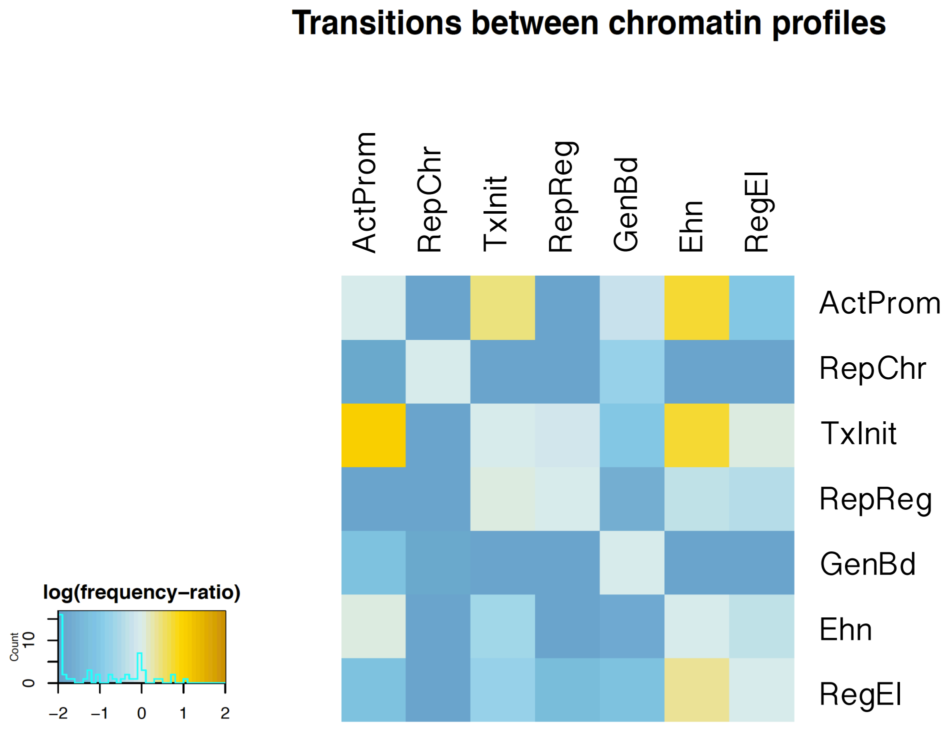


## Figure S4 – Recovery power of poly-adenylation sites in chromatin profiles and single epigenetic marks

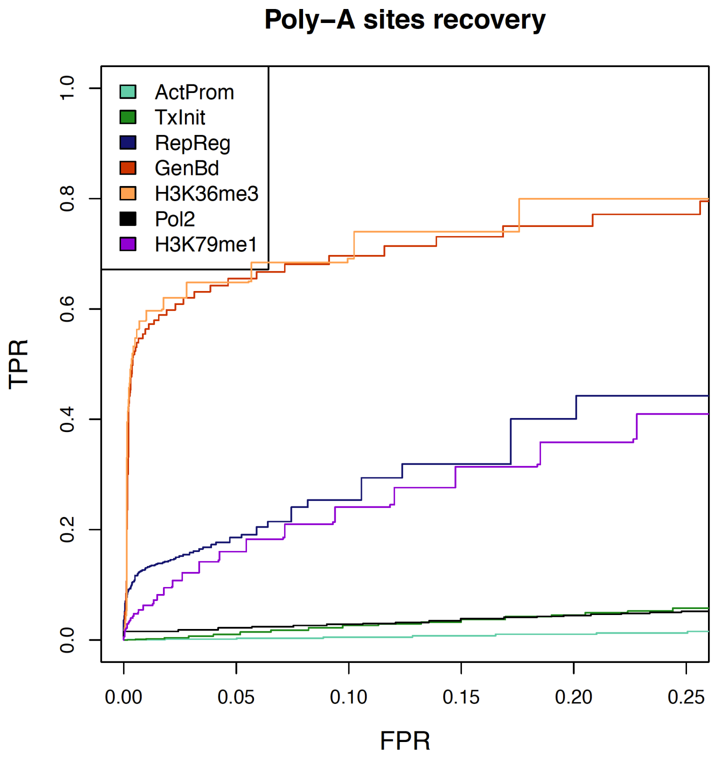

## Figure S5 – Recovery of genomic information using ambiguous profile assignment

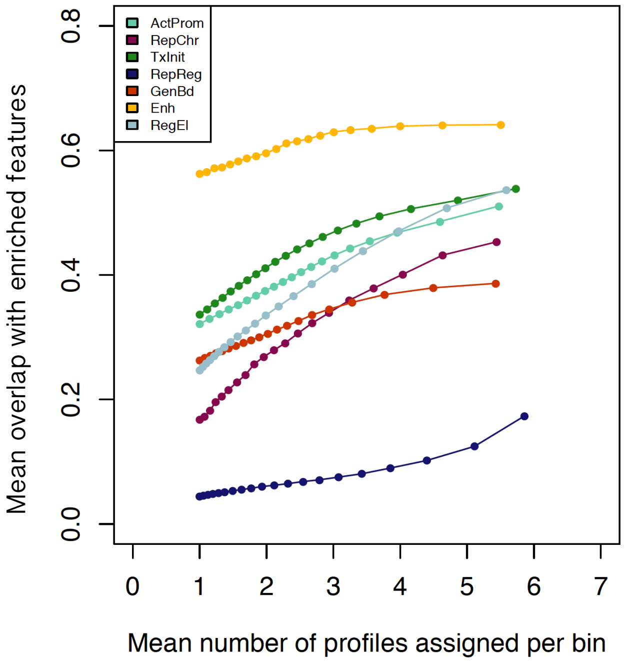

# Figure S6 – Chromatin profile assignment according to expression and distance from the TSS

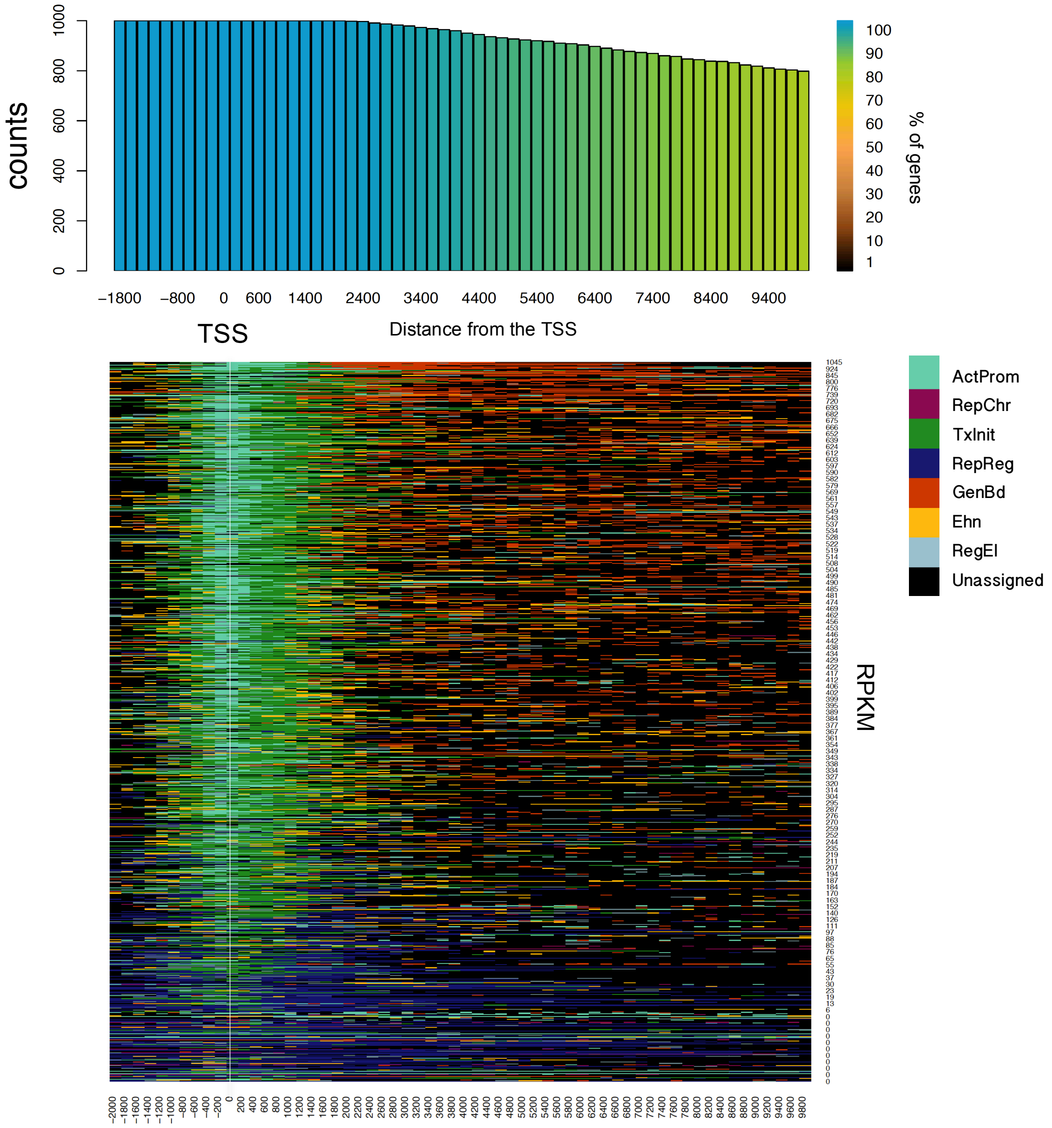

## Figure S7 – Frequency of chromatin profiles according to expression and distance from the TSS

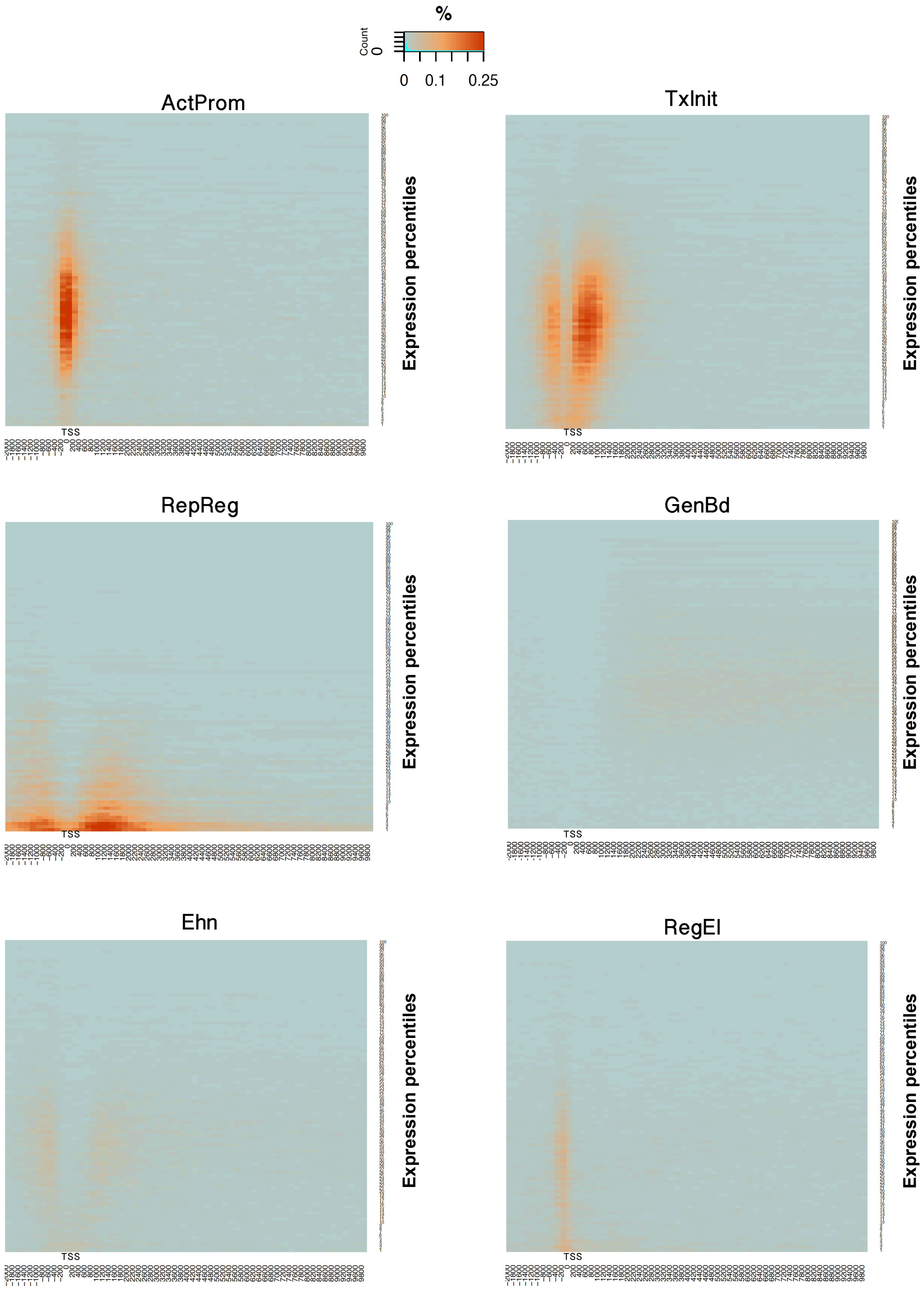

# Figure S8 – Distribution of gene expression in different subclusters

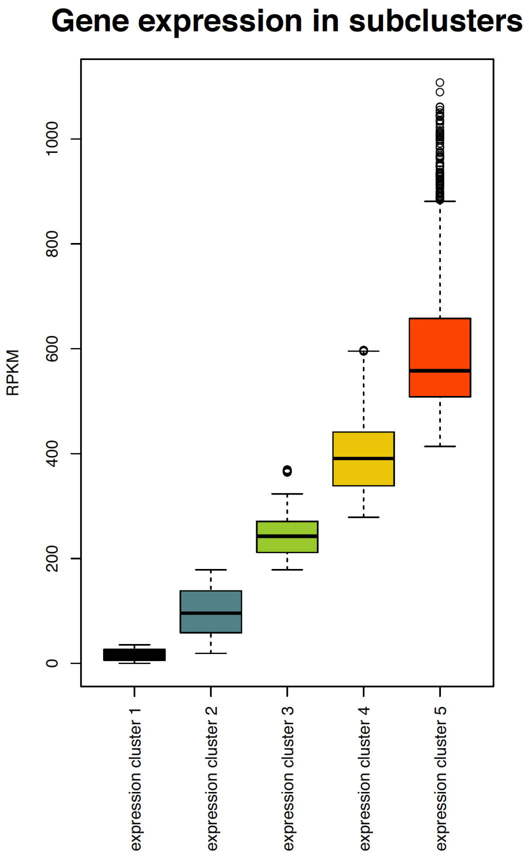

# Figure S9 – Feature enrichment distributions across profiles/states using different methods

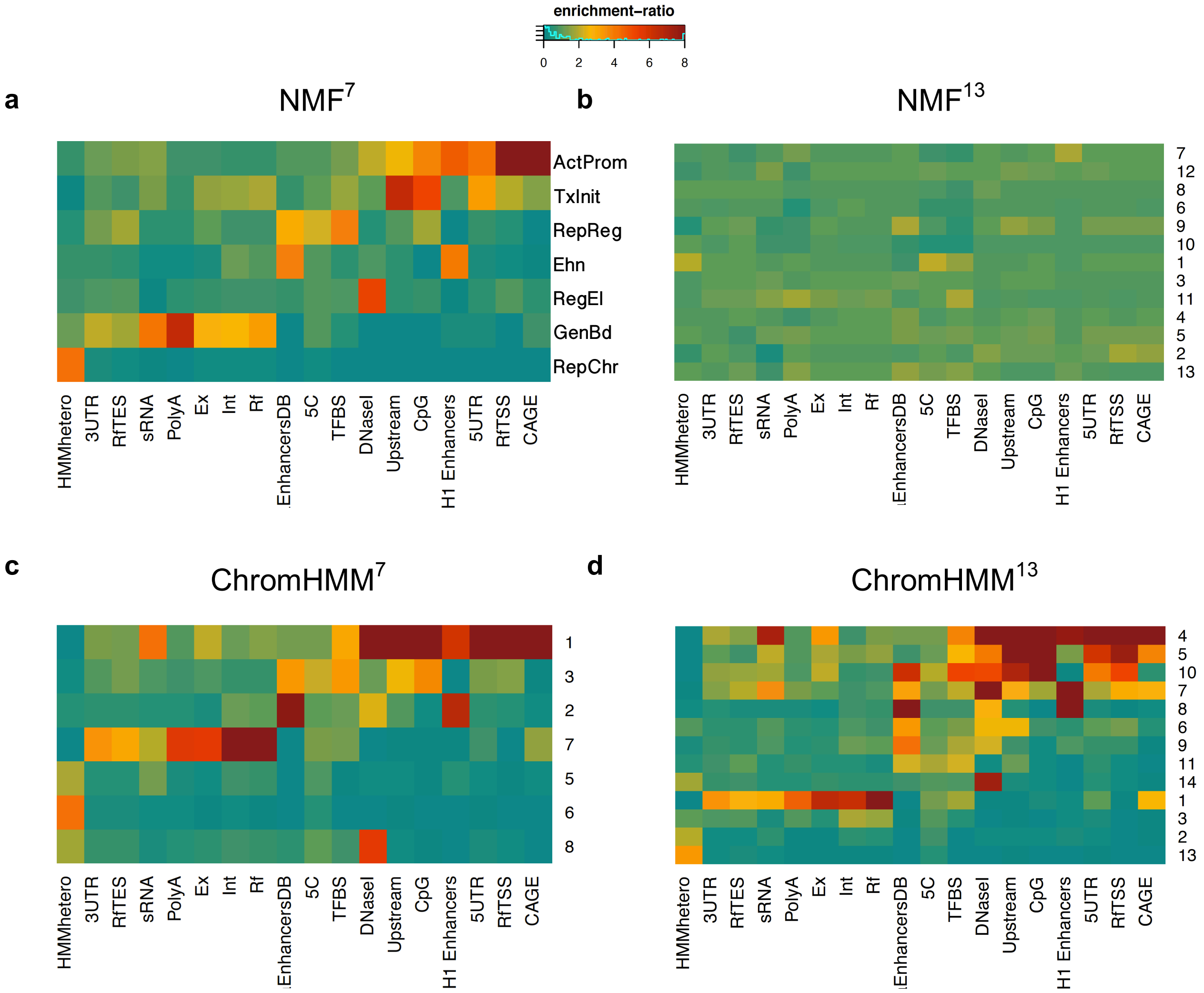

**Figure S10 – Overlap and coverage of functional genomic regions with different combinations of enriched profiles/states**

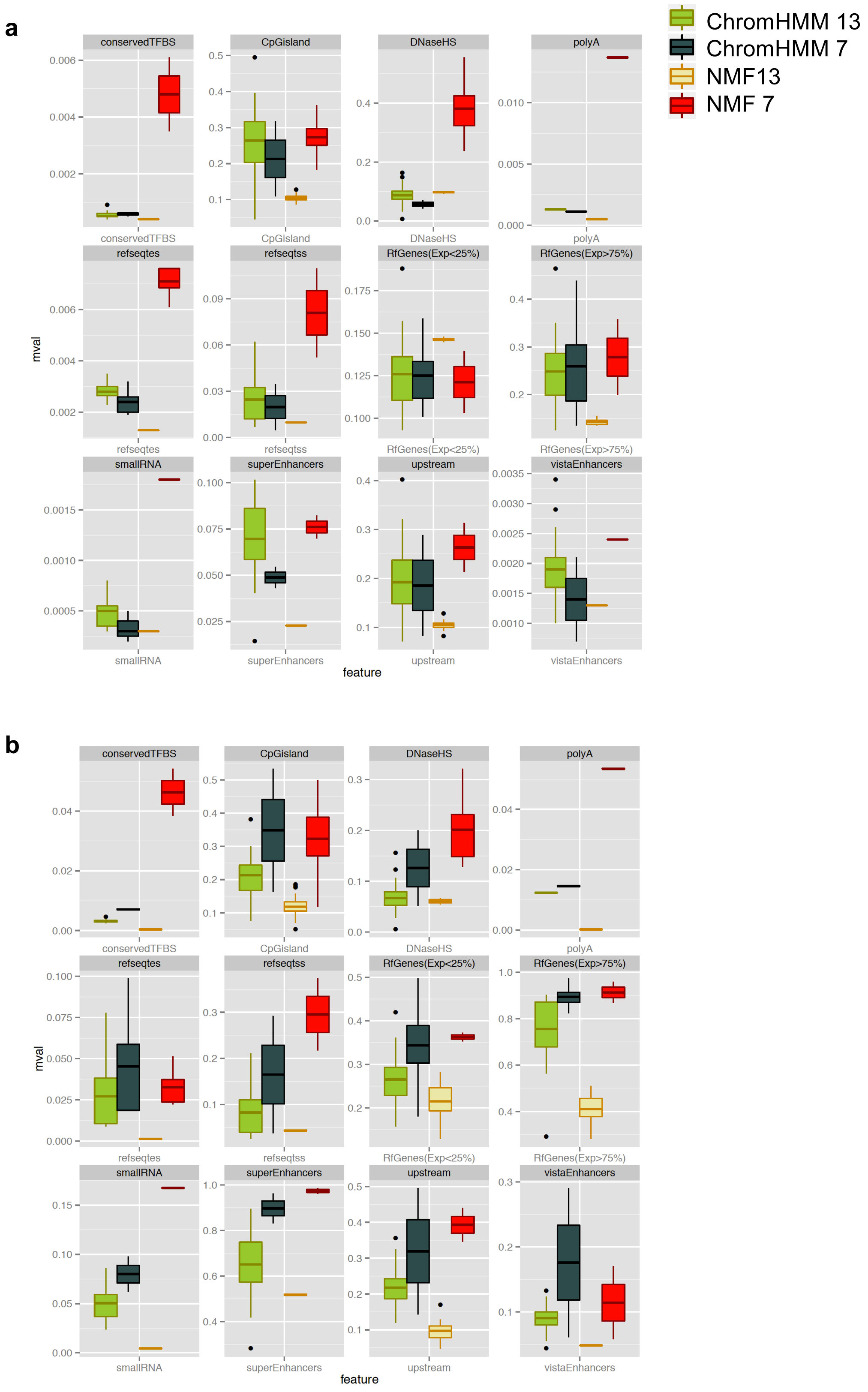

# Table S1 – Combinations of enriched profiles/states used to test the overlap with genomic features in each method.

| | ChromHMM 13 states | | ChromHMM 7 states | | NMF 13 profiles | | NMF 7 profiles | |
|---|---|---|---|---|---|---|---|---|
| Feature | states/profiles | n.combinations | states/profiles | n.combinations | states/profiles | n.combinations | states/profiles | n.combinations |
| 1kb-upstream | 4,5,6,7,10 | 31 | 1,3 | 3 | 2,5,9,12 | 15 | ActProm,TxInit | 3 |
| CpG islands | 4,5,7,10 | 15 | 1,3 | 3 | 2,3,4,5,9,12 | 63 | ActProm,TxInit, RepReg | 7 |
| RefseqTSS(+/-50bp) | 4,5,7,10 | 15 | 1,3 | 3 | 2 | 1 | ActProm,TxInit | 3 |
| RefseqTES(+/-50bp) | 1,4,7,10 | 15 | 1,3,7 | 7 | 11 | 1 | ActProm, RepReg, GenBd | 7 |
| Refseq genes (>75%exp) | 1,3,4,5,9 | 31 | 1,2,7 | 7 | 4,11,12 | 7 | TxInit, GenBd | 3 |
| Refseq genes (<25%exp) | 1,3,4,5,9 | 31 | 1,2,7 | 7 | 4,11,12 | 7 | TxInit, GenBd | 3 |
| PolyAdenylation sites | 1 | 1 | 7 | 1 | 11 | 1 | GenBd | 1 |
| Conserved TFBS | 4,5,9,10,11 | 31 | 1,3 | 3 | 11 | 1 | TxInit, RepReg, | 3 |
| superEnhancers H1 | 4,5,7,8 | 15 | 1,2 | 3 | 7 | 1 | ActProm, Ehn | 3 |
| vistaEnhancersDB | 6,7,8,9,10,11 | 63 | 1,2 | 3 | 13 | 1 | RepReg, Ehn | 3 |
| Dnase-HS | 6,7,8,9,10,11,14 | 127 | 2,3 | 3 | 2,8 | 3 | ActProm,TxInit, RegEl | 7 |
| smallRNAs | 1,4,7 | 7 | 1,7 | 3 | 11 | 1 | GenBd | 1 |

# Figure S11 – NMF and ChromHMM performance in the prediction of distinct functional elements in the genome

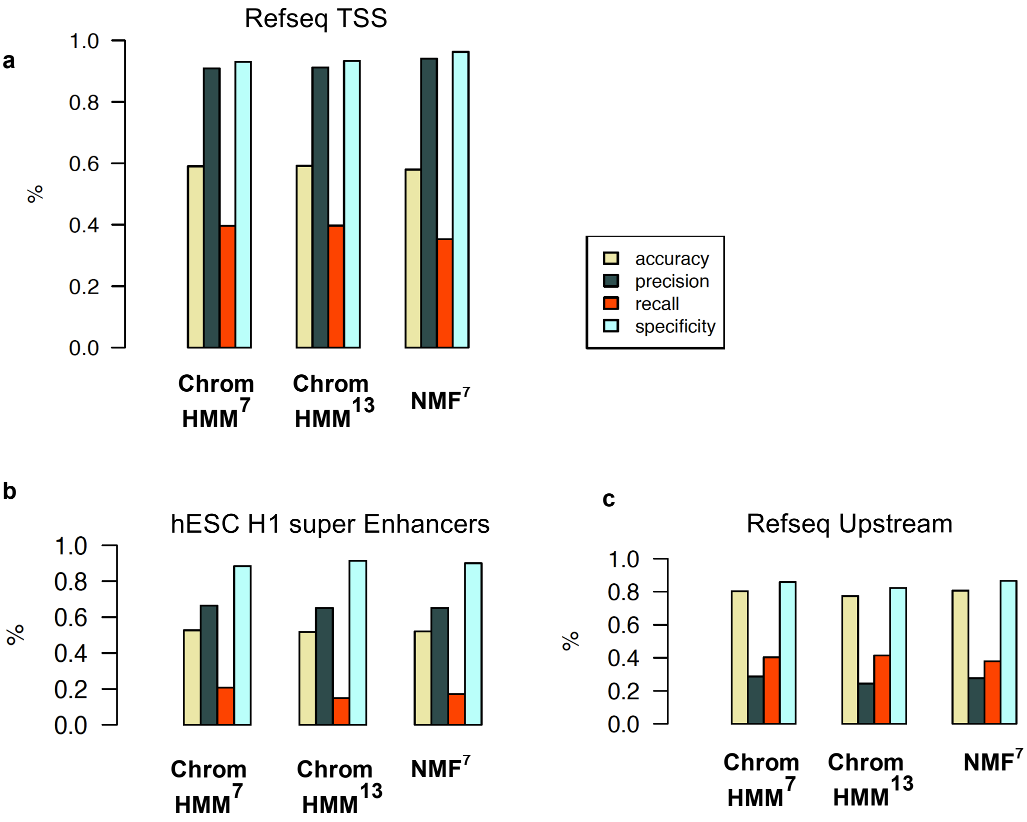

# Figure S12 – Comparison of the NMF chromatin-segmentation approach in different cell lines

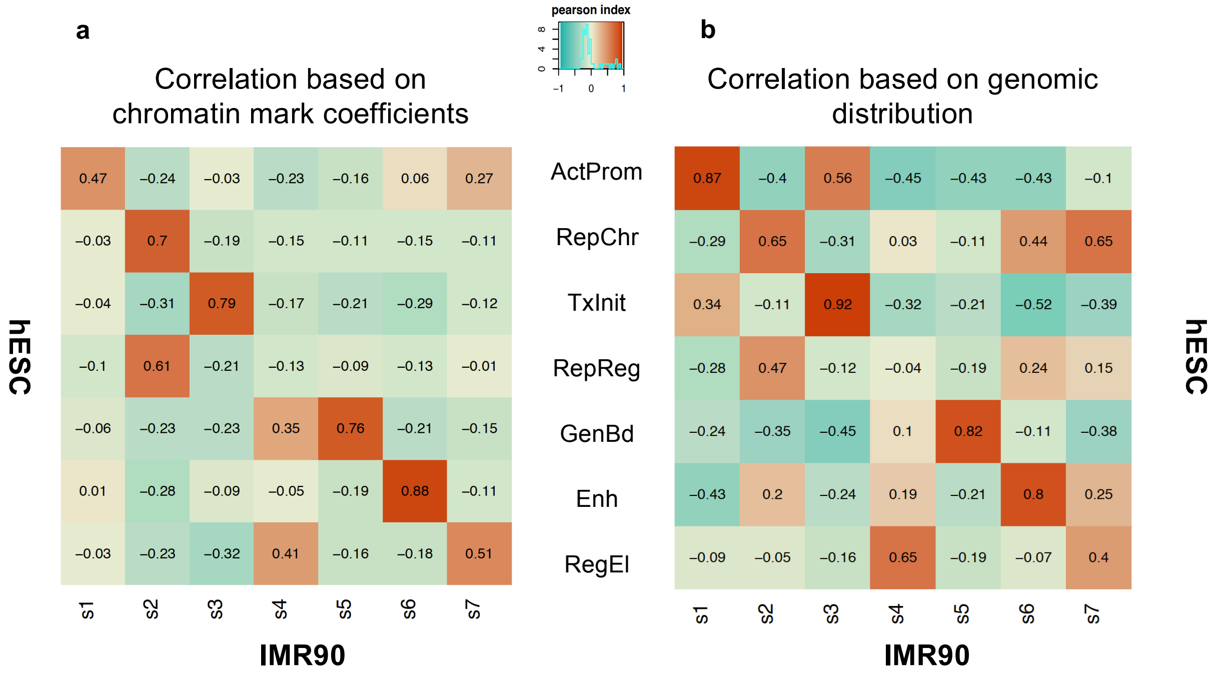

Supplement: Supplementary file 1 — Additional file 1. Supplementary figures and tables with legends (in PDF format). Figure S1: Selection of the best factorization rank. (a) The plot shows the variation of the cophenetic correlation coefficient (on the Y-axis) for both the real (blue) and the random (red) data for increasing values of r. Each point in the real data is obtained after 30 runs of a single NMF analysis using the factorization rank indicated on the X-axis. For the random data, each point indicates the mean cophenetic coefficient obtained by repeating the NMF analysis 30 times at a given factorization rank. The cophenetic coefficient in the real dataset becomes stable at r = 7 remaining at about 0.99 up to r = 11, whilst the stability of the random dataset dramatically drops in the same interval. Within this range, the cophenetic coefficient obtained in the real dataset is more than 4-fold the standard deviation of the coefficients in the random data. b The plot shows the trend of the sparseness in the W (basis) and H (coefficient) matrices over the same range of factorization ranks, in both the real and the random dataset. Figure S2: Significance of chromatin profile enrichment in distinct genomic features. The heatmap shows for each epigenetic profile, the significance of the enrichment compared to different types of genomic features and functional regions of the genome. The significance of the enrichment is assessed using a Fisher’s exact test with a p value of 10−3 as statistical threshold. The color-scale from blue to red indicates the significance of the test as follows: blue: p value > 0.01; purple: 0.01 > p value > 0.001; dark red: 0.001 > p value > 10−5; red: p value < 10−5. A specific biological label is assigned to each profile in order to facilitate its biological interpretation on the basis of the enrichment observed (top-bottom): ActProm = Active Promoter (profile 1); TxInit = Transcription Initiation (profile 3); RepReg = Repressed Regulatory Regions (profile 4); Ehn = Enhan [file 13072_2017_131_MOESM1_ESM.pdf]
