# Supplementary material for: A computational approach for the functional classification of the epigenome
Source: Epigenetics Chromatin. 2017 May 15;10:26. doi: 10.1186/s13072-017-0131-7 (PMC5433140; doi:10.1186/s13072-017-0131-7)
Supplement: Supplementary file 3 — Additional file 3. Archive file (ZIP format) containing all the scripts of the computational pipeline implemented in our work (FGandolfi_pipeline.zip). [file 13072_2017_131_MOESM3_ESM.zip › NMF_pipeline_README.pdf]

NMF processing pipeline; On-line Documentation

———————————————————————————————————————-

Author: Francesco Gandolfi, PhD. Department of Physics, Sapienza Universita' di Roma. P.zza Aldo Moro 00183 Rome, Italy

e-mail: francesco.gandolfi@uniroma1.it

This README contains a detailed description of the scripts and files required to run the processing pipeline mentioned in the manuscript:

"A computational approach for the functional classification of the epigenome"

Francesco Gandolfi and Anna Tramontano

1.Department of Physics, Sapienza University of Rome – P.le A. Moro, 5 – 00185 Rome

2.Istituto Pasteur Italia - Fondazione Cenci Bolognetti, Viale Regina Elena 291, 00161 Rome

——

The pipeline is composed by three different parts that work in sequential manner and are implemented in either bash-scripting or R-environment languages. The pipeline takes, for each sample of a given mark, BAM/BED read alignment files and generates a matrix of normalized coverage signals, which actually represents the input dataset for the NMF algorithm. The last part of the pipeline employs NMF procedure (provided as an R-package) to discovery combinatorial patterns of epigenetic signals.

The 'Processing_Pipeline_Epigenome.ZIP' archive contains all the scripts and files to execute the pipeline.

- bedToNormalizedSignal.sh
- prepareBedAlignment.sh
- binCount2normalizedSignal.R
- wgEncodeDukeMapabilityUniqueness35bp.uniqueMapRegions.bedGraph
- getPooledNormalizedSignal.R
- filterGenomicBins.R

1) SYSTEM COMPATIBILITY

The pipeline currently runs on any Linux operating-system distribution.

Hence, these scripts do not work on a different operating environment (Windows XP/7/10 etc.)

2) PIPELINE REQUIREMENTS

The pipeline scripts require that the following tools, softwares or libraries have been properly installed on the machine in the path /usr/bin to be launched:

- bedtools suite version 2.20 or higher
- samtools version 0.1.19 or higher
- picard-tools 1.95 or higher

- R version 3.0.0 or higher

3) R packages: The following R packages should be properly installed on the machine before the whole pipeline can be run.

- MASS_7.3 or higher
- rtracklayer_1.28.0 or higher
- IRanges_2.2.7 or higher
- GenomicRanges_1.20.5 or higher
- NMF_0.20.1 or higher
- ggplot2_2.1 or higher
- doParallel_1.0.0 or higher
- foreach_1.4.0 or higher

4) PATHS & DIRECTORIES

4.1) Copy and paste all the .R and .sh files (scripting files) in a dedicated directory on the machine. This corresponds to the script directory for the project.

The path for this directory should be indicated in the SCRIPT_DIR variable, which is defined in the first part of the script "bedToNormalizedSignal.sh"

4.2) Set the Project Directory (PROJECT_DIR): To set your Project Directory, open the "prepareBedAlignment.sh" and the "bedToNormalizedSignal.sh" scripts, and change the variable PROJECT_DIR indicating the whole path the directory of the project.

4.3) Create the path specified at 1.2 with the mkdir command.

4.4) Create the Data Directory (DATA_DIR);This is the directory to store primary alignment files for the analysis. To create the Data Directory, move into the directory generated at 1.3, then type: mkdir data. The new Data Directory will be found in the path = PROJECT_DIR/data.

4.5) Move into the Data Directory DATA_DIR and create a subdirectory with the name of cell-line/tissue-type

```
mkdir <CELL-LINE>.
```

4.6) Move into the folder generated at 1.5 and create a subdirectory for each chromatin mark collected for the analysis.

```
mkdir <CHROMATIN_MARK>
```

4.7) Move into each chromatin mark folder generated at 1.6, and create a subfolder for each sample collected for that mark. The new subfolder must be named using the corresponding Sample Accession Number.

```
mkdir <SAMPLEID>
```

4.8) For each sample, put the original mapping (BAM/BED) file into the sample folder (the directory generated at 1.7).

5) INPUT DATA PREPARATION

5.1) "DatasetInfoFile.tsv"

Prior to the execution of the pipeline, a DatasetInfoFile.tsv file must be provided from the user. This file should be a tab-separated text file containing for each sample the following information (columns):

C1: name of the tissue/cell line. THIS ENTRY MUST COINCIDE WITH THE FOLDER NAME GENERATED AT 1.5.

C2: Type of the genomic experimental assay from which data are generated (example: TFBS, openchromatin assay, histone modification etc.)

C3: Name of the chromatin mark represented in the sample. THIS ENTRY MUST COINCIDE WITH THE NAME OF THE CHROMATIN MARK SUBFOLDER GENERATED AT 1.6;

C4: Sample ID (generally, from the Gene Expression Omnibus database). FOR A GIVEN SAMPLE, THIS ENTRY MUST COINCIDE WITH THE NAME OF THE DIRECTORY GENERATED AT 1.7

C5: Name of the Read Alignment File (in BAM or BED format).

C6: Read Alignment Data Type: can be 'raw' if the alignment contains raw sequencing reads or 'processed' if the provided alignment file was already filtered following common processing steps:

a) duplicate reads removal

b) ambiguous reads removal

c) 5'read extension of 200bp

C7: Number of the biological replicate for a given dataset (chromatin mark).

WARNING: Be sure to have the "DatasetInfoFile.tsv" in your own 'data' directory before proceeding with the analysis. Set of chromatin marks for multiple cell lines or tissue types are allowed.

5.2) "chromInfo.txt"

This is a tab-delimited TXT file already provided in the ZIP file. This file contains the following columns:

C1: chromosome name (ex: chr2);

C2: chromosome length;

C3: UCSC Link. This file is already available at the UCSC Genome Browser Download Section (http://hgdownload.soe.ucsc.edu)

5.3) Put these "DatasetInfoFile.tsv" and "chromInfo.txt" files into the Data Directory (PROJECT_DIR/data).

5.4) "wgEncodeDukeMapabilityUniqueness35bp.uniqueMapRegions.bedGraph" This is unique mappability track file already provided in the ZIP file.

-WARNING! To run the pipeline, you have first to specify the path where the Mappability file is stored. To do this, open the "bedToNormalizedSignal.sh" script file and replace the path indicated in the variable MAPPABILITY_TRACK with that of your mappability file. Please see http://hgwdev.sdsc.edu/cgi-bin/hgTrackUi?db=hg18&g=wgEncodeMapability for details about the Mappability track generate for the

ENCODE project. Into the same script, you have also to replace the DATASHEET_SAMPLES_FILE variable (at the beginning of the script) with the path where your **DatasetInfoFile.tsv** is located.

6) PIPELINE EXECUTION

The run the processing pipeline, the different scripts have to be launched in the following order.

6.1) Script 1: prepareBedAlignment.sh

This is the first script required.

The script takes the raw BAM/BED alignment file from a given sample of a single epigenetic mark and performs a number of filtering steps to generate a final BED file called <SAMPLENAME>.processed.bed.

The <SAMPLENAME> term will exactly correspond to the prefix of the original mapping file for that sample. The suffix '.bed' or '.bam' is replaced with the suffix "processed.bed". The output file is generated into the same folder of the original mapping file.

This script performs the following computational steps:

- Duplicate read removal.
- Multi-match read removal.
- Read extension (200bp).

You can execute this script by simply typing the following command:

```
${SCRIPT_DIR}/prepareBedAlignment.sh
```

6.2) Script 2: bedToNormalizedSignal.sh

This is the second script required for the pipeline.

This script takes for each sample of each mark the processed BED file generated in the previous step, and collects the read count distribution from all samples of that mark to calculate the normalized coverage signal estimate. Specifically, the following computational steps are performed in this part of the pipeline:

- Human genome segmentation into 200bp genomic intervals.
- Reconstruction of the read count distribution (assignment of filtered read to the segmented genome).
- Estimation of both the observed and the expected read count distribution in each sample for a given epigenetic mark.
- Estimation of the normalized coverage signal for each chromatin mark.

OUTPUT files:

For each sample analyzed for a given mark, the script generates several output files. All these files are generated in the subfolder specific of that mark, and can be found in the path: PROJECT_DIR/genomic_survey/<CELL-LINE>/<CHROMATIN_MARK>.

The following output files are produced:

- <SAMPLEID>.expectedScore.bg: Tab-delimited file with expected read counts (per bin) in the sample. Columns are: chromosome///bin-start///bin-end///expected counts
- <SAMPLEID>.observedScore.bg: Tab-delimited file with observed read counts (per bin) in the sample. Columns are: chromosome///bin-start///bin-end///observed counts
- <SAMPLEID>.rawCounts.bed: Tab-delimited file with observed read counts (per bin) in the sample. Columns are: chromosome///bin-start///bin-end///observed counts
- <SAMPLEID>.normalizedSignal.bed: Tab-delimited file with normalized coverage signal estimated in the sample. Columns are: chromosome///bin-start///bin-end///normalized read coverage
- expectedScoreSum.bg: Tab-delimited file with expected read counts (per bin) from all samples in the dataset. Columns are: chromosome///bin-start///bin-end///expected read count per sample (1 column per sample)
- observedScoreSum.bg: Tab-delimited file with observed read counts (per bin) from all samples in the dataset. Columns are: chromosome///bin-start///bin-end///observed read count per sample (1 column per sample)
- pooledNormalizedSignal.bed: Tab-delimited file with normalized coverage signal estimated over all samples in the dataset. Columns are: chromosome///bin-start///bin-end///normalized signal
- Files called '<CELL-LINE>_<CHROMATIN_MARK>_allExp_totals.txt' and 'sample_tag_totals.txt' are intermediate files containing the sizes of each sample in the dataset (i.e. the number of mapped reads in each sample), required for the normalized coverage signal estimation.

An additional file called 'hg19binned.200bp.bed' is normally generated into the 'genomic_survey' folder.
This is a tab-separated text file containing the binned version of the human genome hg19. Columns are: chromosome///bin-start///bin-end///.
This is an intermediate file required by the script to generate the raw count distribution for each sample.
You can execute this script typing the following command:

```
${SCRIPT_DIR}/bedToNormalizedSignal.sh
```

-WARNING! Please be sure that the SIGNAL_TRACK variable specified in this script (line 58) reports correctly the set of chromatin marks IN THE SAME ORDER as listed for each cell line in the 'DatasetInfoFile.tsv' , surrounded by round brackets.

6.3) Script 3: filterGenomicBins.R

This script represents the last part of the pre-processing pipeline. The script takes 'pooledNormalizedSignal.bed' files generated for each chromatin mark and combines them together to build a multivariate matrix of normalized signal distributions for all input marks, which also represents the real input of the NMF algorithm. This matrix also corresponds to the V(j,k) matrix described in the manuscript "A computational approach for the functional classification of the epigenome".
The following computational steps are performed in this part of the pipeline:

- Combination of multiple normalized coverage signal distributions (different chromatin marks) into a unique signal matrix V(j,k).
- Filter bins into the V matrix according to a Poisson/Negative-Binomial probability threshold.

-WARNING! Before you run this script, you must enter into the script and change the ‘chipseq.marks’ variable by listing the set of analyzed marks in the same order as reported in the DatasetInfoFile.tsv.

usage:

```
Rscript ${SCRIPT_DIR}/filterGenomicBins.R <CELL-LINE_DIR> <STATDIST> <STAT-THRESHOLD>
```

INPUT arguments:
- <CELL-LINE_DIR>: Character. path to the folder containing all the data generated from scripts n.2 and n.3 for the analyzed cell-line/tissue-type. The folder name must coincide with the name of the tissue/cell-line reported in the “DatasetInfoFile.tsv”/column1.
- <STATDIST>: Character. can be “Poisson” OR “NegativeBinomial”. i.e: the statistical model used to fit the normalized signal distribution of each mark.
- <STAT-THRESHOLD>: Numeric. Tail distribution probability threshold. All genomic bins without at least one epigenetic mark signal under this threshold is treated as not-significant and hence is filtered out. A value between 0.01 and 0.0001 is suggested as best compromise between sensitivity and specificity for this type of count-based data.

OUTPUT files:
- normalizedCompleteMatrix.txt: Tab-delimited text file corresponding to the full (unfiltered) matrix of normalized coverage signals from all chromatin marks. Columns are: chromosome///bin-start///bin-end///normalized signal of epigenetic marks (1 column per mark)
- <STATDIST>.normalizedFilteredMatrix.txt: Tab-delimited text file corresponding to the matrix of normalized coverage signals from all chromatin marks, after filtering with the tail distribution probability threshold specified in <STAT-THRESHOLD>. Columns are: chromosome///bin-start///bin-end///normalized signal of epigenetic marks (1 column per mark). This new matrix represents the input V(j,k) matrix required by NMF.
Both the files are generated into the path specified by <CELL-LINE_DIR>.

6.4) Script 4: runNMFQualityPlots.R

The script performs Non-negative Matrix Factorization analysis to derive combinatorial patterns from integrated epigenetic datasets.
This script also generates a multi-plot PDF which reports a series of quality measurements in order to statistically assess the robustness of the data generated from NMF decomposition.

Prior to NMF analysis, the scripts also applies per-column (i.e.chromatin mark) signal transformation to correct for variability in the ranges of signal from the different mark distributions.

Please refer to the NMF R-package Vignette “An introduction of NMF package” (Renaud Gaujoux, June 2014) for details about the NMF method implemented in R and the different outputs generated in main step of the analysis. The tutorial is available online at http://nmf.r-forge.r-project.org/vignettes/NMF-vignette.pdf

-WARNING! Before you run this script, you must enter into the script and change the ‘chipseq.marks’ variable by listing the set of analyzed marks in the same order as reported in the DatasetInfoFile.tsv.

usage:

```
Rscript ${SCRIPT_DIR}/runNMFQualityPlots.R <NORMALIZED_FILTERED_MATRIX_FILE>
<TRANSF_FUNCTION> <FACT_RANK_RANGE> <NRUNS> <OUTPUT_DIR> <FACT_RANK_ANALYZED>
```

INPUT arguments:

- <NORMALIZED_FILTERED_MATRIX_FILE>: Character. This is the path to the matrix of normalized coverage signals from all chromatin marks,

i.e. the <STATDIST>.normalizedFilteredMatrix.txt (or <STATDIST>.normalizedRandomMatrix.txt if you test random data).

This also corresponds to the main output file generated by the function “**filterGenomicBins.R**”

- <TRANSF_FUNCTION>: Character. It can be “raw” OR “sigmoid” OR “invhyperbolic”. This is the parameter indicating the mathematical function used for the signal transformation.

“raw” = the normalized data are not transformed; “sigmoid”= each column is transformed used a sigmoid function such that all values will be flat between 0 and 1; “invhyperbolic”= each column is transformed used inverse hyperbolic sine function (very similar to logarithmic data conversion).

- <FACT_RANK_RANGE>: Character. This parameter should be provided in the form “A,B” (quoting included) where A is the smallest Factorization Rank value the NMF analysis starts from and B is the highest Factorization Rank value. IMPORTANT: All the Factorization Rank values within this range will be included in the analysis. It means that a different NMF analysis will be repeated for each rank value in from A to B. You can also launch the NMF analysis with a unique Factorization Rank value by simply specifying “R,R” , where R indicates the Factorization Rank desired.

- <NRUNS>: Number of NMF runs used to perform a single NMF analysis. We suggest to set a minimum of 30 runs to achieve reasonable cluster stability from the NMF procedure.

- <OUTPUT_DIR>: Character. It defines the path of the directory where the output of the NMF will be placed.

- <FACT_RANK_ANALYZED>: Numeric. This is the Factorization Rank value for which results will be extracted from the NMF object and used to reconstruct the profile-bin assignment of the chromatin segmentation process. For the Factorization Rank specified in this argument, the corresponding H-matrix

(i.e. the matrix of coefficients of each profile across input chromatin marks) and the Average-Signal matrix will be generated as heatmaps in PDF format.

IMPORTANT: Set this parameter to 0 to suppress all the additional analyses focused on the NMF results from a specific factorization rank.

OUTPUT files:

- <TRANSF_FUNCTION>.<STATDIST>.nmf.distributions.RData: An .RData file containing the R-object 'estim'. This is a LIST object which stores all the results of the NMF analysis. The list is structured such that each element in the list corresponds to an object of class 'NMFfit', which collects NMF fitting data from a single NMF analysis using a single factorization rank. (i.e. the number of elements in the list will correspond to the number of factorization ranks that will be assessed, as specified in the argument FACT_RANK_RANGE). Please note that the STATDIST argument, which is used here as a label for the .RData file name exactly corresponds to the string STATDIST previously specified for the function "filterGenomicsBins.R" (Script nr.3).

Please, refer to the "NMF" R-package tutorial at https://cran.r-project.org/web/packages/NMF/NMF.pdf for a detailed description of the 'NMFfit' class R-object and how data are organized within the NMFfit structure.

- <TRANSF_FUNCTION>.<STATDIST>.quality.test.NMF.pdf: This PDF is a collection of three plots with a number of quality measures to describe robustness of the NMF data decomposition over the range of factorization ranks assessed.

i) First (leftmost) plot: Cophenetic Correlation Coefficient (Brunet et al. "Metagenes and molecular pattern discovery using matrix factorization". Proceedings of the National Academy of Science of United States of America, 2004) in function of the different factorization rank tested. The Cophenetic Correlation Coefficient is defined as the correlation between the sample distances from the consensus matrix and the distances obtained by its hierarchical clustering, thus providing a measure of cluster robustness and stability using a specific factorization rank.

ii) Second (middle) plot: Sparsity index (Hoyer et al. "Non-negative Matrix Factorization with sparseness constrains", The Journal of Machine Learning Research, 2004) for both the H (Coefficient) and the W (Weight) matrix generated in the single NMF analysis, obtained for each factorization rank. Hoyer's Sparsity takes values between 0 (all vector elements of the matrix are equal) and 1 (single non-zero component).

iii) Third (rightmost) plot: Residual sum of squares (RSS), between the target matrix (V) and the NMF estimate, extracted from the NMFfit object for each factorization rank in the range FACT_RANK_RANGE.

-<TRANSF_FUNCTION>.<STATDIST>.fr<FACT_RANK_ANALYZED>.profileAssignment.bed (OPTIONAL, only with <FACT_RANK_ANALYZED> higher than 0): This is a text (tab-delimited) file in BED format reporting the assignment of chromatin profiles for each genomic bin analyzed by NMF. The following data are reported along the columns(left-right):chromosome///bin-start///bin-end///bin-id(in the form 'chrom:bin-start_bin-end')///chromatinProfileAssigned///strand (always '.').

The file is generated with chromosomes (and bins) sorted by increasing genomic coordinate.

- <TRANSF_FUNCTION>.<STATDIST>.fr<FACT_RANK_ANALYZED>.coeffMatrix.pdf (OPTIONAL, only with <FACT_RANK_ANALYZED> higher than 0): a color-scale heatmap representing H (the coefficient matrix) generated from NMF using the factorization rank (i.e. number of chromatin profile for signal decomposition). The heatmap provides a easier interpretation of the different chromatin profile composition. Each cell P{x,y}in the heat map is colored according the contribution (coefficient) of the profile X (indicated in the row) to the chromatin input mark Y (indicated in the column).
- <TRANSF_FUNCTION>.<STATDIST>.fr<FACT_RANK_ANALYZED>.coeffMatrix.txt (OPTIONAL, only with <FACT_RANK_ANALYZED> higher than 0): A TXT file of the H-coefficient matrix represented in the 'coeffMatrix.pdf' file, reported in tabular format.
-<TRANSF_FUNCTION>.<STATDIST>.fr<FACT_RANK_ANALYZED>.meanChromatinSignal.pdf (OPTIONAL, only with <FACT_RANK_ANALYZED> higher than 0): a color-scale heat map showing the mean normalized read coverage of a given chromatin mark (column) over all genomic bins assigned to a given profile (row).

6.5) Script 5: getRandomizedData.R

The script uses the 'randomize' built-in function from NMF-R package to produce a random version of the original dataset used for the analysis.

It produces a random normalized signal matrix whose factorization serves as a reference for selecting the best factorization rank, that does not overfit the data.

Given an input matrix V, randomization is performed by permutating independently the entries in each column of V, generating a new 'random' data matrix of the same size.

Please, refer to the "NMF" R-package tutorial at https://cran.r-project.org/web/packages/NMF/NMF.pdf for a detailed description of the method.

usage:

```
Rscript ${SCRIPT_DIR}/getRandomizedData.R <NORMALIZED_FILTERED_MATRIX_FILE> <OUTPUT_DIR>
```

INPUT arguments:

- <NORMALIZED_FILTERED_MATRIX_FILE>: Character. This is the path to the matrix of normalized coverage signals from all chromatin marks.
- <OUTPUT_DIR>: Character. The entire path to the folder where the output data will be located. A 'random' sub-folder will be added to this path to better distinguish output files generated for the random dataset from those of the real dataset. You will find this output file into the 'random' sub-folder of OUTPUT_DIR.

OUTPUT files:

- <STATDIST>.normalizedRandomMatrix.txt: A tab-delimited text file which corresponds to the random matrix generated by the script.

Please note that the STATDIST label refers to the same STATDIST term specified as argument to 'filterGenomicBins.R'.

6.6) Script 6: overlapRealAndRandomData.R

-WARNING! Use this script only once NMF has been already tested on both the real and the random dataset using the same set (range) of factorization ranks. The script takes quality parameters obtained from both the real and the random data matrix and merges them together to generated a multi-plot PDF file showing overlapping distributions for the two dataset for each quality measure previously analyzed in the script '**runNMFQualityPlots.R**'

usage:

```
Rscript ${SCRIPT_DIR}/overlapRealAndRandomData.R <NORMALIZED_FILTERED_MATRIX_FILE>
<SAMPLE_NMF_RESULTS> <NORMALIZED_RANDOM_MATRIX_FILE> <RANDOM_NMF_RESULTS> <OUTPUT_DIR>
```

INPUT arguments:

- <NORMALIZED_FILTERED_MATRIX_FILE>: Character. This is the path to the matrix of normalized coverage signals from all chromatin marks.
- <SAMPLE_NMF_RESULTS>: Character. The path to the .RData file containing results from the NMF analysis performed on the original dataset.
- <NORMALIZED_RANDOM_MATRIX_FILE>. Character. This is the path to the random matrix of the normalized coverage signals from all chromatin marks in the dataset. Should corresponds to the entire path of the <STATDIST>.normalizedRandomMatrix.txt file, generated by Script 5.
- <RANDOM_NMF_RESULTS>: Character. The path to the .RData file containing results from the NMF analysis performed on the random dataset.
- <OUTPUT_DIR>: Character. The entire path to the folder where the output data will be located.

OUTPUT files:

- <TRANSF_FUNCTION>.<STATDIST>.Real+Random.quality.test.NMF.pdf: This PDF is a collection of three plots with a number of quality measures to describe robustness of the NMF data decomposition over the range of factorization ranks assessed. In each plot, changes of a specific quality parameter are represented for both the real (blue/purple) and the random (red/green) dataset.

i) First (leftmost) plot: Cophenetic Correlation Coefficient (Brunet et al. "Metagenes and molecular pattern discovery using matrix factorization". Proceedings of the National Academy of Science of United States of America, 2004) in function of the different factorization rank tested. The Cophenetic Correlation Coefficient is defined as the correlation between the sample distances from the consensus matrix and the distances obtained by its hierarchical clustering, thus providing a measure of cluster robustness and stability using a specific factorization rank.

ii) Second (middle) plot: Sparsity index (Hoyer et al. “Non-negative Matrix Factorization with sparseness constrains”, The Journal of Machine Learning Research, 2004) for both the H (Coefficient) and the W (Weight) matrix generated in the single NMF analysis, obtained for each factorization rank. Hoyer’s Sparsity takes values between 0 (all vector elements of the matrix are equal) and 1 (single non-zero component).
iii) Third (rightmost) plot: Residual sum of squares (RSS), between the target matrix (V) and the NMF estimate, extracted from the NMFfit object for each factorization rank in the range FACT_RANK_RANGE.

7) ANALYSIS GUIDELINE : Suggested procedure for the estimation of the best factorization rank

A common approach to find the best factorization rank is to try NMF in a pre-defined range of r values, estimate a quality measure of the results, and select the best value of r according to this quality criteria (Gaujoux et al. “A flexible R package for non-negative Matrix Factorization”, BMC Bioinformatics 2010).
In our work, we use the Cophenetic Correlation Coefficient to measure the robustness of the data obtained with a given factorization rank value. To statistically assess changes in the Cophenetic Coefficient, we strongly recommend to repeat the analysis on the random dataset and then compare the two distributions (real and random) to identify significant changes in the cluster stability. Specifically, we suggest to take the factorization rank above which the cluster stability (i.e. the cophenetic coefficient) of the real dataset started being significantly higher than that in the random (e.g. the smallest value of r at which the cophenetic coefficient from the real dataset starts increasing and the cophenetic coeff. in the random starts decreasing should be a reasonable choice).
According to this approach, we strongly recommend to run the scripts the whole pipeline on the basis of the following outline:

7.1) Integration/Pre-processing of the data

STEP#1: launch prepareBedAlignment.sh
STEP#2: launch bedToNormalizedSignal.sh
STEP#3: launch filterGenomicBins.R

7.2) Non-negative Matrix Factorization: statistical assessment

STEP#4: launch getRandomizedData.R
STEP#5: launch runNMFQualityPlots.R on the real dataset (with <FACT_RANK_ANALYZED> set to 0 and <FACT_RANK_RANGE> = r(min),R(max))
STEP#6: launch runNMFQualityPlots.R on the random dataset (with <FACT_RANK_ANALYZED> set to 0 and <FACT_RANK_RANGE> = r(min),R(max))
STEP#7: launch overlapRealAndRandomData.R

7.3) Non-negative Matrix Factorization: analysis

STEP#8: launch runNMFQualityPlots.R on the real dataset (with <FACT_RANK_ANALYZED> set to K, where K is the factorization rank value selected after STEP#7).
